# Internalization and accumulation of model lignin breakdown products in bacteria and fungi

**DOI:** 10.1186/s13068-019-1494-8

**Published:** 2019-07-03

**Authors:** Meghan C. Barnhart-Dailey, Dongmei Ye, Dulce C. Hayes, Danae Maes, Casey T. Simoes, Leah Appelhans, Amanda Carroll-Portillo, Michael S. Kent, Jerilyn A. Timlin

**Affiliations:** 1 Department of Bioenergy and Defense Technologies, Sandia National Laboratories, Albuquerque, NM 87123 USA; 2 Department of Nano and Bio-Sensors, Sandia National Laboratories, Albuquerque, NM 87123 USA; 3 Department of Nanobiology, Sandia National Laboratories, Albuquerque, NM 87123 USA; 4 Department of Organic Materials Science, Sandia National Laboratories, Albuquerque, NM 87123 USA; 5 Present Address: Section of Gastroenterology, New Mexico VA Health Care System, Albuquerque, NM 87108 USA

**Keywords:** Lignin, Bioconversion, Transport, Single-cell analysis, Mass spectrometry, Mono-aryl, Di-aryl

## Abstract

**Background:**

Valorization of lignin has the potential to significantly improve the economics of lignocellulosic biorefineries. However, its complex structure makes conversion to useful products elusive. One promising approach is depolymerization of lignin and subsequent bioconversion of breakdown products into value-added compounds. Optimizing transport of these depolymerization products into one or more organism(s) for biological conversion is important to maximize carbon utilization and minimize toxicity. Current methods assess internalization of depolymerization products indirectly—for example, growth on, or toxicity of, a substrate. Furthermore, no method has been shown to provide visualization of depolymerization products in individual cells.

**Results:**

We applied mass spectrometry to provide direct measurements of relative internalized concentrations of several lignin depolymerization compounds and single-cell microscopy methods to visualize cell-to-cell differences in internalized amounts of two lignin depolymerization compounds. We characterized internalization of 4-hydroxybenzoic acid, vanillic acid, *p*-coumaric acid, syringic acid, and the model dimer guaiacylglycerol-beta-guaiacyl ether (GGE) in the lignolytic organisms *Phanerochaete chrysosporium* and *Enterobacter lignolyticus* and in the non-lignolytic but genetically tractable organisms *Saccharomyces cerevisiae* and *Escherichia coli*. The results show varying degrees of internalization in all organisms for all the tested compounds, including the model dimer, GGE. *Phanerochaete chrysosporium* internalizes all compounds in non-lignolytic and lignolytic conditions at comparable levels, indicating that the transporters for these compounds are not specific to the lignolytic secondary metabolic system. Single-cell microscopy shows that internalization of vanillic acid and 4-hydroxybenzoic acid analogs varies greatly among individual fungal and bacterial cells in a given population. Glucose starvation and chemical inhibition of ATP hydrolysis during internalization significantly reduced the internalized amount of vanillic acid in bacteria.

**Conclusions:**

Mass spectrometry and single-cell microscopy methods were developed to establish a toolset for providing direct measurement and visualization of relative internal concentrations of mono- and di-aryl compounds in microbes. Utilizing these methods, we observed broad variation in intracellular concentration between organisms and within populations and this may have important consequences for the efficiency and productivity of an industrial process for bioconversion. Subsequent application of this toolset will be useful in identifying and characterizing specific transporters for lignin-derived mono- and di-aryl compounds.

**Electronic supplementary material:**

The online version of this article (10.1186/s13068-019-1494-8) contains supplementary material, which is available to authorized users.

## Introduction

Lignin, cellulose, and hemicellulose are the three main biopolymers comprising lignocellulosic biomass. Lignin is the most abundant aromatic plant component in terrestrial ecosystem and is 15–35% of the dry weight of lignocellulosic biomass [1]. Lignin is a complex heteropolymer composed of three hydroxycinnamyl alcohol monomers, each differing in the number of methoxy groups. These three monomers combine in various ways through radical polymerization during lignification, forming a variety of carbon–oxygen and carbon–carbon bonds, resulting in *p*-hydroxyphenyl H, guaiacyl G, and syringyl S phenylpropanoid units once polymerized [2]. The percentage of the three monomer types varies depending on the biomass source. Consequently, breakdown of this complex polymer results in a large range of chemical species and molecular weights. This poses a particular challenge to lignin valorization as separation and purification of individual compounds from a complex lignin breakdown stream is a daunting task.

Biological conversion holds great promise for accommodating the diversity of lignin breakdown products by virtue of the ability of organisms to funnel carbon from various molecules into central metabolism [3–6]. In both nature and industrial lignocellulosic biorefineries, lignin depolymerization can occur extracellularly via a host of oxidases and peroxidases secreted by fungi and bacteria and by non-enzymatic reactions utilizing hydrogen peroxide radicals [7]. These extracellular lignin biodegradation processes result in a mixture of monomeric and oligomeric aromatic hydrocarbons [8–10]. Low molecular weight lignin breakdown products are then transported into lignolytic bacteria and certain fungi and are subsequently metabolized [5, 11, 12], although evidence for internalization in white rot fungi, including *P. chrysosporium*, has not been presented to date. It is widely believed that fungi play the primary role in breaking down lignin into low molecular weight species and that bacteria mineralize a large fraction of the low molecular weight material [3, 13, 14]. Indeed, a wide range of bacteria have been identified that can grow on aromatic compounds. In early studies, growth and toxicity provided important insights. Early studies included growth of *Pseudomonas putida* (strain FK2) on guaiacylglycerol-β-coniferyl ether [15], growth of mixed bacterial cultures on monomers (such as guaiacol, vanillic acid, and coumaric acid) [16], lignin-like dimers [16, 17] and tetramers [18, 19], and growth of *Xanthomonas* strain 99 on lignin oligomers ranging up to 1000 g/mol [20]. These studies suggested that lignin monomers, dimers and perhaps even higher-order oligomers are transported across bacterial cell membranes. However, it is unclear whether the target molecules were internalized directly or broken down extracellularly before internalization because direct measurement of internal concentrations was not performed. While aromatic acids can diffuse across membranes when present in high concentrations [21, 22], active transport systems are also present in many bacteria and fungi to make use of the low (μM) concentrations of these compounds in soils.

Engineering anabolic pathways for efficient conversion of this carbon into useful products or chemical intermediates is currently an area of active research [23, 24]. Optimizing transport of lignin breakdown products into organisms for biological conversion may also improve conversion efficiency. Depolymerization of lignin is an energy intensive process that inevitably results in a broad molecular weight distribution. It will most likely be very difficult and expensive to generate a stream composed entirely of monomers [25]. It is therefore important to understand and potentially expand the range of compounds that can be internalized and metabolized in organisms used for biological conversion. Further, in optimizing a biological conversion process, the rates of internalization may need to be tuned so that uptake rate is neither limiting nor so high as to be detrimental to the host [26–28].

In this work, quantitative methods for directly measuring intracellular concentrations of lignin breakdown products in microbes were developed. These methods were applied to investigate internalization of four common monomeric lignin depolymerization products and the model lignin dimer, GGE into two lignolytic organisms, *Enterobacter lignolyticus* SCF1 (*E. lignolyticus*) and *Phanerochaete chrysosporium* (*P. chrysosporium*) and two non-lignolytic organisms, *Escherichia coli* (*E. coli*) *and Saccharomyces cerevisiae* (*S. cerevisiae*). *P. chrysosporium* is a white rot basidiomycetes that possesses the ability to degrade lignin and is one of the primary wood-rotting organisms present in nature [28]. Despite being the focus of lignin breakdown studies for many decades [29–31], the range of lignin breakdown products that are internalized by this organism has not yet been characterized. Similarly, *E. lignolyticus*, a soil bacterium, has been shown to assimilate lignin compounds [32], but the range of compounds taken up has yet to be studied comprehensively. The non-lignolytic organisms were included because they can be readily genetically modified for subsequent studies of specific transporters. *E. coli* is not able to break down lignin, nor grow on most aromatic acids [21], but it is able to mineralize some aromatic compounds, including some aromatic acids (phenylacetic acid, 4-hydroxyphenylacetic acid, phenylpropionic acid, 3-hydroxyphenylpropionic acid, and 3-hydroxycinnamic acid), and possesses several MFS transporters for these compounds [22]. This, along with the fact that aromatic acids are generally lipophilic and have structural similarities to amino acids, leads to the possibility that these molecules could be transported passively or perhaps by promiscuous sugar transporters or aromatic amino acid transporters at high (mM) concentrations. *S. cerevisiae* is commonly used for industrial fermentation, and toxicity of certain phenolic compounds is an important practical concern [27]. Thus, in addition to providing background data for subsequent studies of specific transporters engineered into these organisms, direct measurements of internal concentrations of these compounds in *E. coli and S. cerevisiae* are relevant for developing and testing strategies for reducing toxicity in industrial processes involving these organisms.

**Fig. 1:**
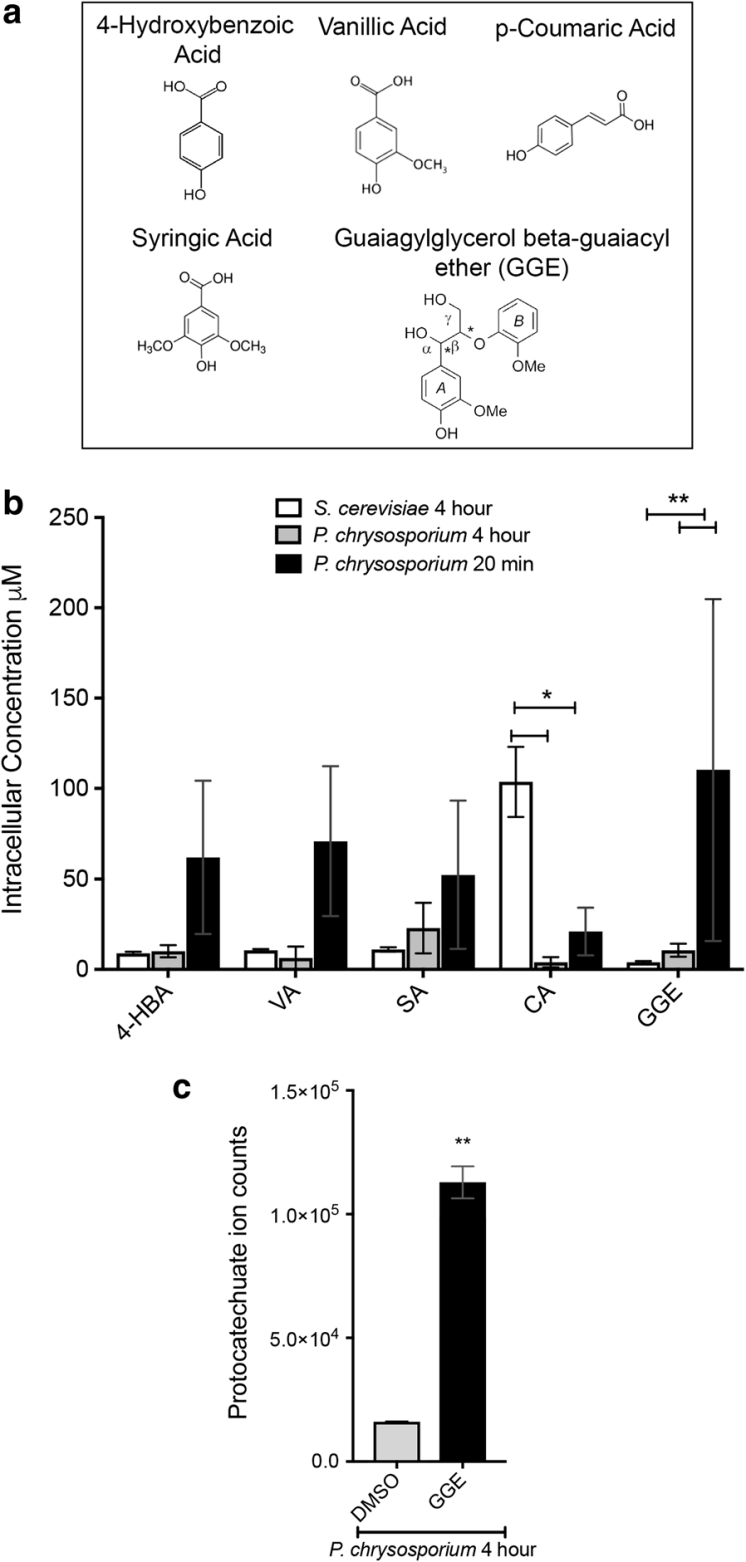
Internalization of lignin fragments into lignolytic and non-lignolytic fungi. **a** Chemical structures of mono- and di-lignols tested in internalization assays. **b** Relative lysate concentrations (µM) obtained from QToF LC/MS analysis of mono- and di-lignols in *S. cerevisiae* (white bars) and *P. chrysosporium* cell lysates after 4 h (gray bars) or 20 min (black bars) of lignol internalization. Error bars show the standard deviation of three biological replicates. *Indicates *p* value of < 0.0461, **Indicates *p* value < 0.004 following two-way ANOVA analysis. **c** Average ion counts for protocatechuate (GGE metabolite) in *P. chrysosporium* cell lysates incubated with DMSO or GGE for 4 h. Error bars show standard deviation of two biological replicates. **p value 0.0022 by *t* test

Using mass spectrometry and single-cell click chemistry-enabled microscopy, we quantitatively compare internalized amounts of lignin breakdown compounds at both the population and single-cell level after an incubation time spanning the log-phase growth timeframe of the microbes. We chose 4-hydroxybenzoic acid (4-HBA), syringic acid (SA), vanillic acid (VA), *p*-coumaric acid (CA) and the di-aryl compound, guaiacylglycerol beta-guaiacyl ether (GGE) (Fig. 1a) because they have been identified in the literature as products from several lignin breakdown processes [1, 5, 6]. We demonstrate that both lignolytic and non-lignolytic bacterial and fungal organisms internalize these compounds to varying degrees. The two lignolytic organisms, *E. lignolyticus* and *P. chrysosporium*, internalize GGE at statistically higher levels as compared to the non-lignolytic organisms, *E. coli* and *S. cerevisiae*, respectively. We lastly demonstrate that both chemical inhibition of oxidative phosphorylation and glucose starvation result in decreased internal concentrations of vanillic acid in *E. coli*, indicating that internalization of VA in *E. coli* requires energy. Our results demonstrate the utility of this toolset for direct measurement of internal concentrations of mono- and di-aryl compounds. Characterizing transport will provide important fundamental understanding to advance biological conversion of lignin breakdown products.

## Results

### Internalization of lignin fragments into lignolytic and non-lignolytic fungi

Five compounds representative of lignin breakdown products were tested for internalization and accumulation within the lignolytic white rot fungus *P. chrysosporium* and the non-lignolytic fungus *S. cerevisiae*. *P. chrysosporium* spores were inoculated into fungal media and grown on microcrystalline cellulose for 5 days to induce lignolytic conditions [33]. *S. cerevisiae* cells were grown in synthetic media to mid-log phase. The mono- and di-aryl compounds were then added to the media at a concentration of 4 mM for 4 h. For direct comparison between the two organisms, the 4-h time point was chosen to encompass log-phase growth in *S. cerevisiae.* The *P. chrysosporium* mycelia and *S. cerevisiae* cells were harvested, and lysates were prepared for LC/MS analysis. Relative lysate concentrations were calculated from ion counts using spiked lysate standards (Additional file 1: Figure S1 and Additional file 2: Figure S2). Control lysates prepared from cells incubated with a vehicle control (DMSO) confirmed that the peak of interest for each compound was only present in the lysates when the mono-/di-aryls were added to the media (Additional file 1: Figure S1 and Additional file 2: Figure S2). The LC/MS results show that all mono-aryl compounds were internalized to some degree in *P. chrysosporium* at the 4-h time point, with lysate concentrations ranging from 3.9 µM (CA) to 22.9 µM (SA) (Fig. 1b, gray bars). The di-aryl GGE also accumulated to a substantial level in the *P. chrysosporium* cells at the 4-h time point at an average concentration of 10.6 µM (Fig. 1b). A 20-min time point for internalization was also collected to determine if metabolism of any of the five compounds was occurring in *P. chrysosporium* by 4 h. If compounds are metabolized over time we expected to see greater accumulation at shorter incubation times. Consistent with this expectation, at the 20-min time point 2- to 11-fold higher concentrations were observed for the various aryl compounds tested (Fig. 1b, black bars). The di-aryl GGE was strongly enriched at 20 min (110.3 µM) versus 4 h (10.6 µM). Further suggesting that *P. chrysosporium* might metabolize GGE, the levels of the GGE metabolite protocatechuate detected by LC/MS were enriched sixfold over DMSO controls in *P. chrysosporium* lysates after 4-h of incubation (Fig. 1c).

LC/MS of *S. cerevisiae* cell lysates revealed uptake of each of the mono-aryl compounds at low levels, with the exception of *p*-coumaric acid (CA) (Fig. 1b, white bars). Among the four mono-aryls, CA accumulated much higher levels in *S. cerevisiae* than the other compounds, reaching an average lysate concentration of 103.7 µM (Fig. 1b). Accumulation of GGE in *S. cerevisiae* was low and near the detection limit.

### Single-cell microscopy of lignin fragment internalization in fungi

**Fig. 2:**
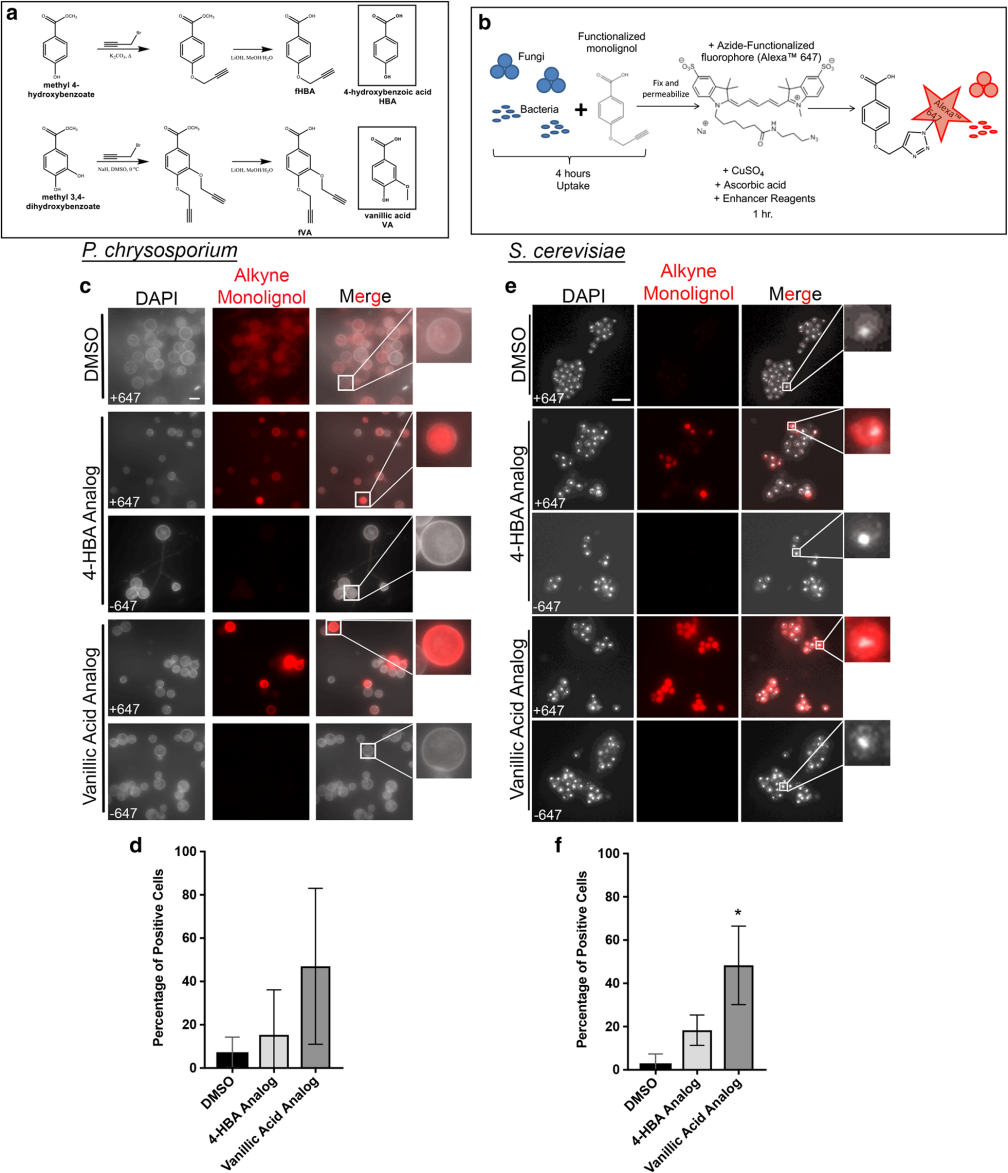
Single-cell microscopy of lignin fragment internalization in fungi. **a** Chemical synthesis pathway of mono- and di-functionalized 4-HBA (fHBA) and vanillic acid (fVA), respectively. **b** Schematic of click chemistry-mediated fluorescent labeling of bacteria and fungi. Imaging samples were paired with LC/MS studies and done in biological triplicate. **c** Representative images of *P. chrysosporium* cells following 4 h of internalization with functionalized lignols, fixation, and AlexaFluor™ 647 labeling. **d** Quantification of experiment in **c**. **e** Representative images of *S. cerevisiae* cells following 4 h of internalization with functionalized lignols, fixation, and AlexaFluor™ 647 labeling. **f** Quantification of experiment in **e**. Error bars in **d** and **f** represent standard deviation of three biological replicates. *Indicates *p* value of 0.0146 by Kruskal–Wallis test. Scale bars in **c** and **e** represent 5 µm

To visualize internalization at the individual cell level and to assess variability from cell to cell, we utilized a click chemistry-assisted labeling scheme followed by confocal fluorescence microscopy to quantify intracellular levels of the mono-aryl compounds in single cells with minimal modification to the compounds. We functionalized two of the mono-aryl compounds for compatibility with click chemistry by adding an alkyne group. 4-HBA and vanillic acid were selected as representative compounds to functionalize as they were amenable to alkyne functionalization. The synthesis of the alkyne-functionalized compounds is shown in Fig. 2a and is described fully in “Materials and methods” section. Attempts to synthesize a mono-functionalized VA analog in which only the para-hydroxy group is functionalized with the alkyne to mirror the steric and electronic profile of the methoxy group in vanillic acid resulted in intramolecular cyclization (Additional file 3: Figure S3) rendering the product unusable for click chemistry. A di-functionalized vanillic acid analog with two click sites for fluorophores to bind represents the closest analog and was used for these studies. This compound can also be considered as an analogue to protocatechuic acid (PCA), although PCA was not included in this study. *P. chrysosporium* cells were grown for 5 days in lignolytic conditions and then allowed to internalize the alkyne-functionalized 4-HBA and vanillic acid compounds at 4 mM for the established 4-h time point. *S. cerevisiae* cells were grown to mid-log phase and then similarly exposed to the alkyne-functionalized compounds for 4 h. DMSO was used as a vehicle negative control. Cells were then fixed, permeabilized and click-labeled with AlexaFluor™ 647 (Fig. 2b). Single-cell analysis was performed using DAPI stain to identify cell locations, and then corresponding AlexaFluor™ 647 signal was measured in those locations. A threshold intensity above background was set to accommodate the moderate levels of autofluorescence for these cells types and to conservatively identify positive cells. The single-cell image analysis results are summarized in Additional file 4: Figure S4 for three biological replicates for each organism and condition. *P. chrysosporium* is a member of the Basidiomycota division, and it contains septate hyphae, spore-bearing cells called chlamydospores, and thin-walled basidiospores [34]. We observed strongest AlexaFluor™ 647 signal above background in the basidiospores and focused our *P. chrysosporium* single-cell analysis on those cells. The mono-alkyne 4-HBA analog was taken up by 15.3% of the basidiospores, and the di-alkyne vanillic acid analog was taken up by 47.0% of the spores (Fig. 2c, d). In the *S. cerevisiae* cells, the mono-functionalized 4-HBA analog was taken up by 18.3% of cells and the di-functionalized vanillic acid analog was taken up by 48.3% of cells (Fig. 2e, f). Additional file 5: Figure S5 shows the mean intensities for positive cells indicating substantial variations in intracellular concentrations at the individual cell level (Additional file 5: Figure S5A and B).

### Comparison of compound accumulation in lignolytic and non-lignolytic states of *P. chrysosporium*

**Fig. 3:**
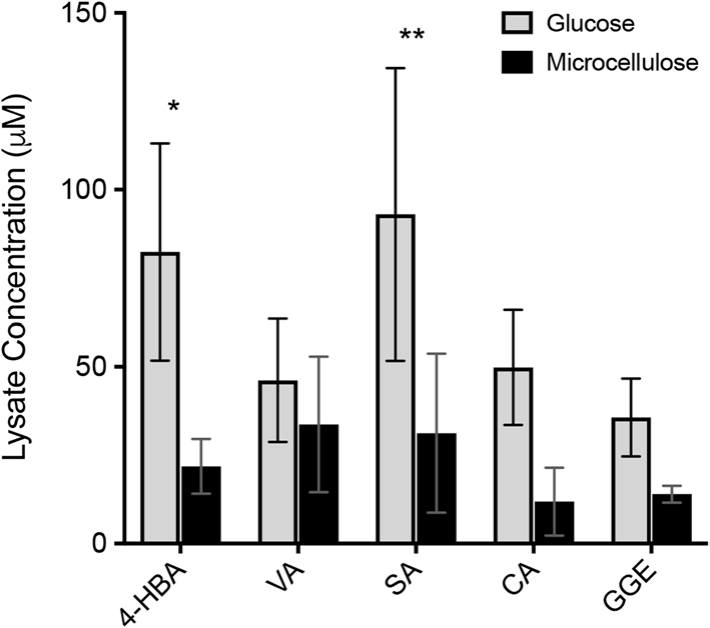
Comparison of compound accumulation in lignolytic and non-lignolytic states of *P. chrysosporium*. Relative lysate concentrations (µM) obtained from QToF LC/MS analysis of mono- and di-lignols in lysates of *P. chrysosporium* grown in the presence of either glucose (non-lignolytic, gray bars) or microcellulose (lignolytic, black bars). 20-min time point is shown. Error bars represent the standard deviation of three biological replicates. *Indicates *p* value of 0.01, **Indicates *p* value 0.0085 following two-way ANOVA analysis

We also tested whether internal concentrations of the aryl compounds are modulated according to the lignolytic state of the fungus. *P. chrysosporium* mycelia were grown in the presence of either glucose (non-lignolytic) or microcrystalline cellulose (lignolytic) for 5 days [33]. Manganese peroxidase activity was detected in the latter condition verifying the lignolytic state, whereas no manganese peroxidase activity was detected in the former condition (Additional file 6: Figure S7). Mycelia were then exposed to the aryl compounds for 20 min in the media and processed for LC/MS analysis. At this time point, high accumulation of each compound was detected in the non-lignolytic state as well as in the lignolytic state (Fig. 3). However, in the lignolytic state, the intracellular levels of most compounds were roughly twofold less than in the non-lignolytic state.

### Evidence of lignin fragment catabolism

**Fig. 4:**
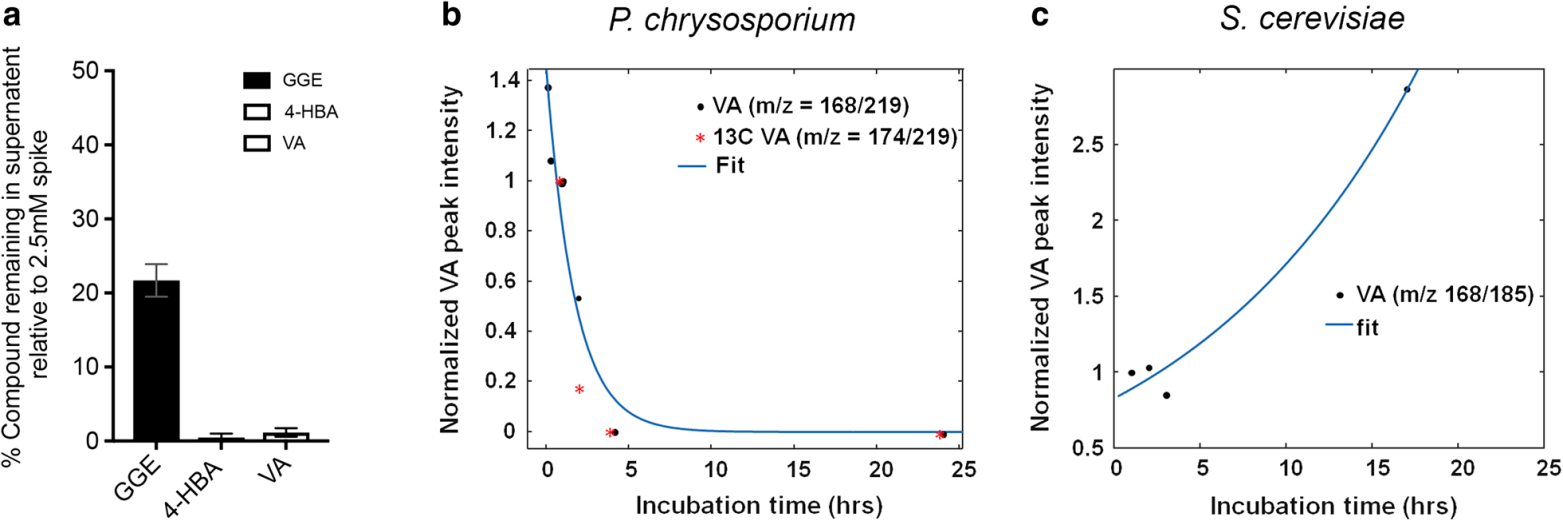
Evidence of lignin fragment catabolism in fungi. **a** HPLC results measuring the percentage of compounds remaining in PBS following 72 h of internalization by *P. chrysosporium*. Percentages were calculated using a 2.5 mM spiked sample. **b** ASAP-MS results of vanillic acid (*m/z* = 168.02) and 6C13 vanillic acid (*m/z* = 174.03) in *P. chrysosporium* lysate following a time course of internalization from 1 to 10 h. Vanillic acid and 6C13 vanillic acid were measured in separate biological replicates. No counts above background were detected at *m/z* = 168.02 and *m/z* = 174.03 in absence of addition of these compounds. **c** ASAP-MS results of vanillic acid peak intensity in *S. cerevisiae* following a time course of internalization from 1 to 17 h. A single biological replicate is shown for **c**

As described in the previous section, in *P. chrysosporium* lysates with micromolar levels of internalized di-aryl GGE, a possible metabolite, protocatechuate, was also identified at levels well above the control [3]. This is consistent with internal catabolism of this di-aryl in *P. chrysosporium* (Fig. 1c). Protocatechuate has been suggested as a metabolite of GGE through the vanillin/vanillate pathway in *Sphingomonas* sp. *SYK6* [3]. Hydroxypropriovanillone (HPV) has been identified as a breakdown product of GGE in sphingomonads [3, 35]. Additional evidence of catabolism of GGE, 4-hydroxybenzoic acid, and vanillic acid in *P. chrysosporium* is shown through substantial depletion of the mono- and di-aryl compounds from PBS after 72-h incubation with the lignolytic mycelia as measured by HPLC (Fig. 4a), although we cannot rule out extracellular modification of the compounds as well. The ability of *P. chrysosporium* to metabolize vanillic acid over time was also investigated. *S. cerevisiae* and *P. chrysosporium* cells were incubated with 0.1% vanillic acid for times ranging from 5 min to 24 h. Cells were then harvested and analyzed by atmospheric solid analysis probe–mass spectrometry (ASAP-MS) (Additional file 7: Figure S6). Over the time course, vanillic acid levels in *P. chrysosporium* lysate decreased over several hours, in a pattern consistent with metabolism (Fig. 4b). In contrast, vanillic acid levels in *S. cerevisiae* lysate increased monotonically with incubation time, strongly suggesting little or no metabolism of that compound as expected for this non-lignolytic organism (Fig. 4c).

### Internalization of lignin fragments into lignolytic and non-lignolytic bacteria

**Fig. 5:**
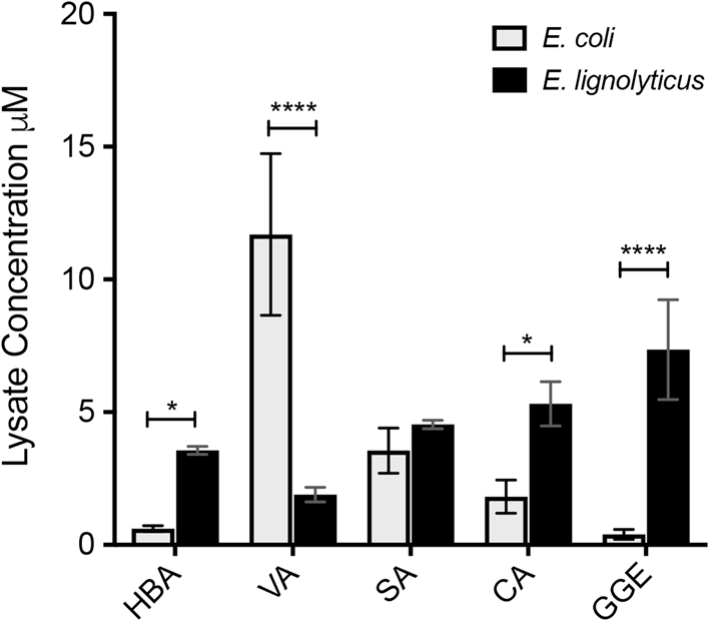
Internalization of lignin fragments into lignolytic and non-lignolytic bacteria. Relative lysate concentrations of mono- and di-lignols (µM) obtained from QToF LC/MS analysis of *E. coli* (gray bars) versus *E. lignolyticus* (black bars) lysates following 4 h of internalization. Error bars represent standard deviation of three biological replicates. *Indicates *p* value of < 0.0372, ****indicates *p* value < 0.0001 following two-way ANOVA analysis

We next applied LC/MS to measure internalization in bacteria. The same five compounds were tested for internalization and accumulation in a lignolytic bacterium (*E. lignolyticus*) and a non-lignolytic bacterium (*E. coli*). *E. lignolyticus* cells were grown to mid-log phase in nutrient broth. The mono- and di-aryl compounds were then added to the media at a concentration of 4 mM for 4 h to study internalization during log-phase growth. Cells were then lysed and prepared for analysis by LC/MS (Fig. 5a). Control lysates prepared from cells incubated with a vehicle control (DMSO) confirmed that the peak of interest for each compound was only present in the lysates when the mono-/di-aryls were added to the media (Additional file 8: Figure S8). Untreated *E. lignolyticus* cultures were grown alongside these to mid-log phase, lysed using 70% ethanol, and spiked with known concentrations of the mono- and di-aryl compounds of interest for use as calibration standards (Additional file 8: Figure S8). Intracellular accumulation occurred for all mono-aryl compounds and the di-aryl compound GGE in *E. lignolyticus* (Fig. 5a, black bars). This indicates that dimers as well as monomers can enter the cells and raises the possibility that even larger oligomers may also be internalized. Metabolites of GGE, specifically, guaiacol (G), protocatechuate (PC), and 2-(2-methoxyphenoxy)-1,3-propanediol (2MPP), were detected at much higher levels (6–8*x*) compared with the DMSO control when *E. lignolyticus* was grown in minimal media. However, these compounds were detected at only background levels when *E. lignolyticus* was grown in nutrient broth (Additional file 9: Figure S9).

We also measured internalization of the same compounds in non-lignolytic *E. coli*, a useful host for studying engineered transporters [21]. Cultures grown to mid-log phase in minimal M9 media internalized all five compounds (Fig. 5a, gray bars). For all compounds except vanillic acid, the lysate concentrations in *E. coli* were lower than for *E. lignolyticus* (Fig. 5a). Far less GGE and HBA (0.39 µM and 0.61 µM, respectively) were detected in cell lysates of *E. coli* than for *E. lignolyticus* (7.35 µM and 3.56 µM, respectively). Vanillic acid accumulated to much higher levels in *E. coli* than in *E. lignolyticus*, reaching lysate concentrations of nearly 12 µM on average (Fig. 5a).

### Single-cell analysis of lignin fragment internalization in bacteria

**Fig. 6:**
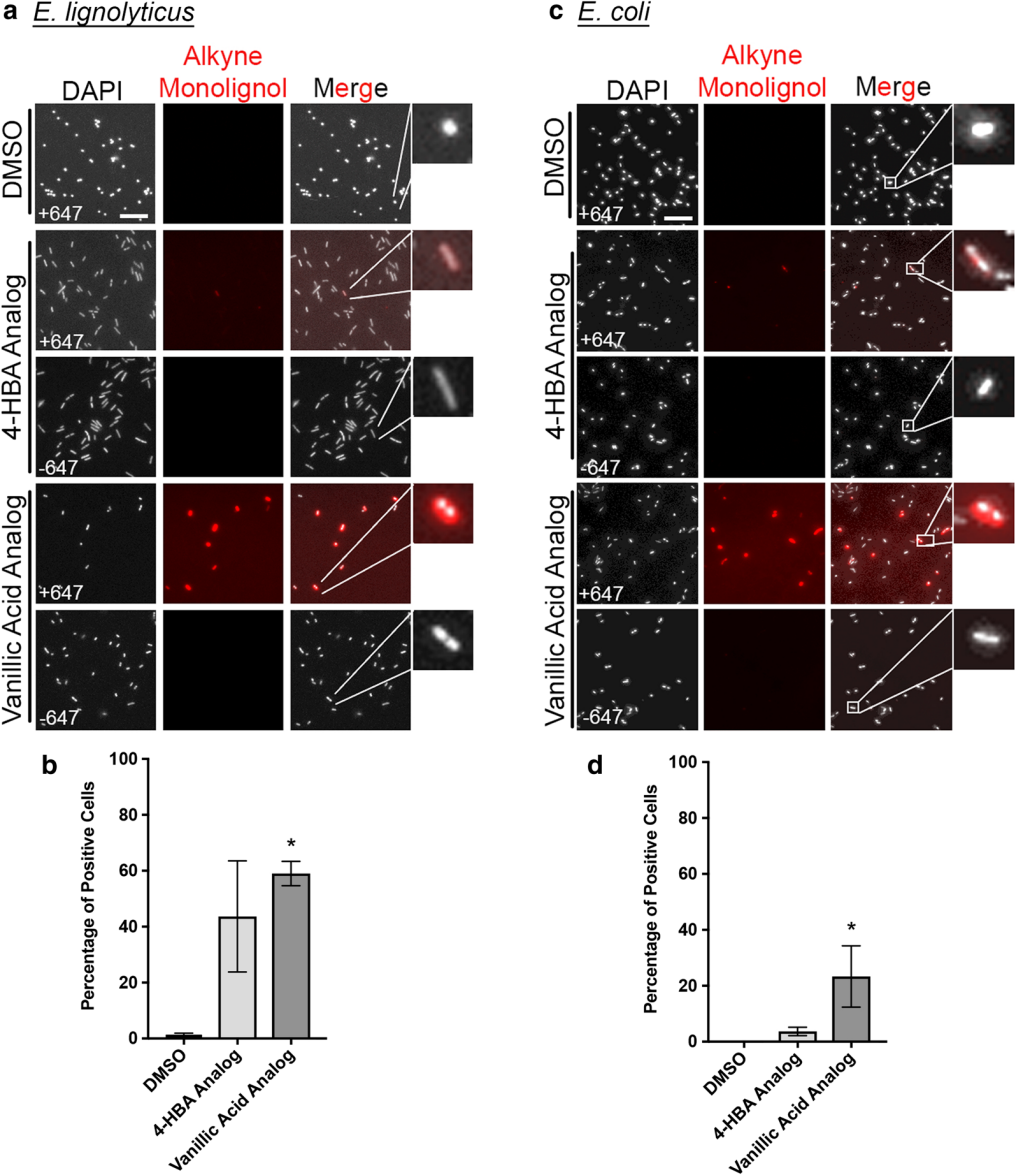
Single-cell microscopy of lignin fragment internalization in bacteria. **a** Representative images of *E. lignolyticus* cells following 4 h of internalization with functionalized lignols, fixation, and AlexaFluor™ 647 labeling. **b** Quantification of experiment in **a**. **c** Representative images of *E. coli* cells following 4 h of internalization with functionalized lignols, fixation, and AlexaFluor™ 647 labeling. **d** Quantification of experiment in **c**. Scale bars in **a** and **c** represent 5 µm. Bars in **b**, **d** are the average of three biological replicates, and error bars represent standard deviation of these replicates. *Indicates *p* value of 0.0262 for **b** and 0.0127 for **d** by Kruskal–Wallis test

To determine internalization variability from cell to cell in bacteria, we again utilized the click chemistry-assisted labeling scheme followed by confocal fluorescence microscopy to visualize intracellular levels of the mono-aryl compounds in single cells with minimal modification to the compound. *E. lignolyticus* or *E. coli* cells were grown to mid-log phase then exposed to the alkyne-functionalized 4-HBA or vanillic acid analogs at 4 mM concentration in the media for 4 h. Cells treated with DMSO were used as a vehicle negative control. Cells were then fixed, permeabilized, and click-labeled with AlexaFluor™ 647. Fluorescence microscopy followed by single-cell analysis was performed using DAPI stain to identify cell locations and then AlexaFluor™ 647 signal was measured in those locations (Fig. 6a, c). Cells were counted as positive for uptake of the compound as described in “Materials and methods” section to provide robust quantitation of intracellular levels of the compounds in individual cells above the background in the control images (Fig. 6b, d). In *E. lignolyticus*, the mono-functionalized 4-HBA analog was internalized by 43.7% of the cells, and the di-functionalized vanillic acid analog was internalized by 59.0% of the cells (Fig. 6a, b). Comparatively, in *E. coli*, the 4-HBA analog was internalized by 3.7% of cells and the vanillic acid analog was internalized by 23.3% of cells (Fig. 6c, d). Similar to the fungi, a large range of intensities between cells was observed for each compound, suggesting large cell-to-cell variations in intracellular concentration (Additional file 5: Figure S5C and D). *E. lignolyticus* clearly demonstrates a bimodal distribution of intensities for both 4-HBA and VA analogs with a substantial fraction of the positive cells having a low level of fluorescence and a smaller fraction having much stronger and more variable signal intensities. Interestingly, incubation of the *E. lignolyticus* cells with the functionalized analogs also resulted in an increase in cell aspect ratio (Additional file 10: Figure S10).

### Evidence for active transport

**Fig. 7:**
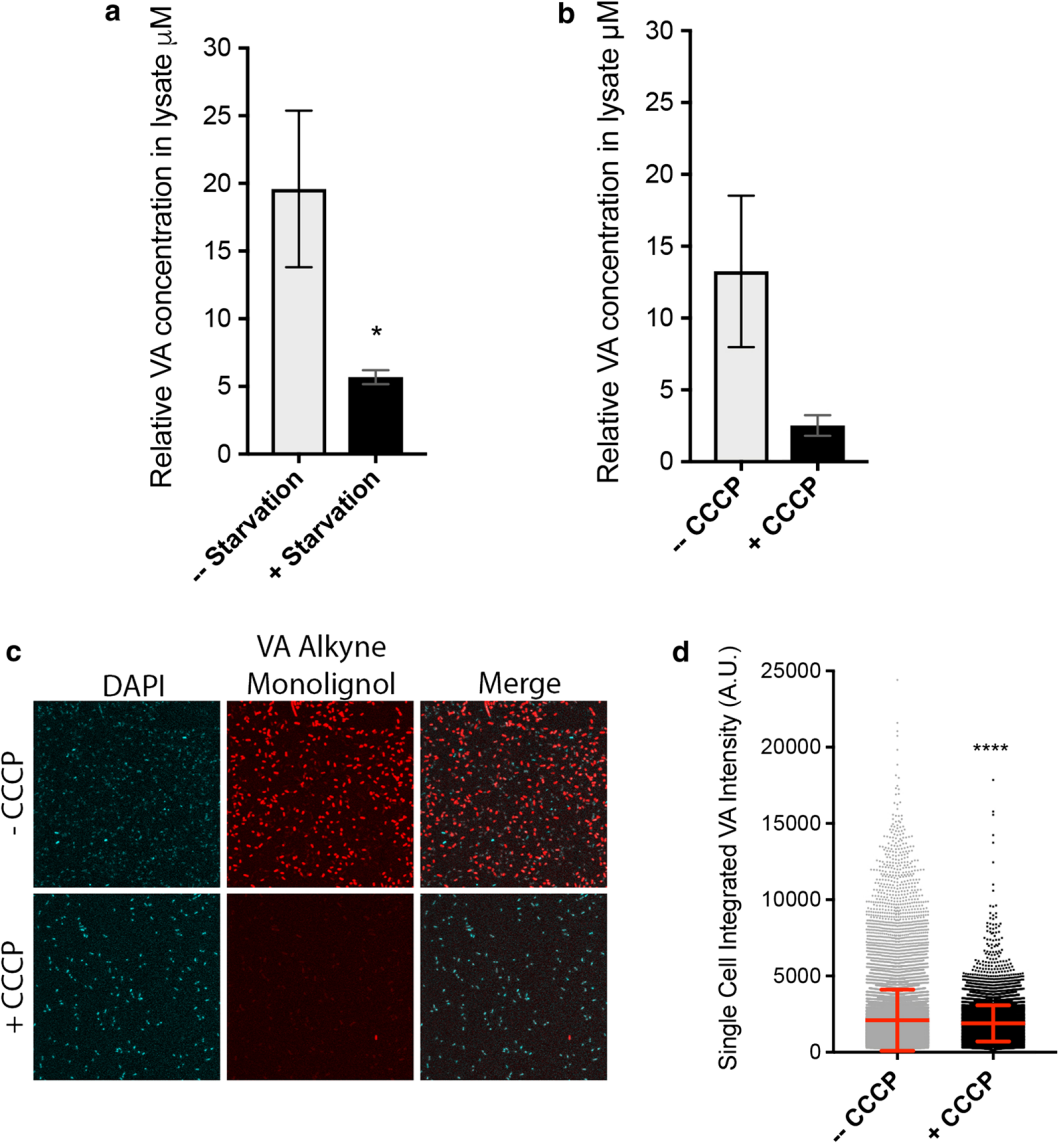
Lignin fragment internalization requires energy in bacteria. **a** TQD-MS results showing relative VA concentrations in *E. coli* lysates generated from cells glucose starved for 4-h internalization timeframe. Three biological replicates are shown. **b** TQD-MS results showing relative VA concentrations in *E. coli* lysates following CCCP or vehicle control treatment for 4 h. Two biological replicates are shown. **c** Representative images of *E. coli* ± CCCP for 4 h internalization of the alkyne-modified VA mono-lignol (fVA) followed by fixation and AlexaFluor™ 647 labeling. **d** Single-cell microscopy analysis of fVA in *E. coli* cells treated with CCCP or vehicle control for 4 h. Single-cell integrated intensities are graphed, red lines mark mean, and whiskers mark standard deviation of > 10,000 cells per condition, representing three biological replicates. *Indicates *p* value of 0.014, and ****indicates *p* value of < 0.0001 by a *t*-test

Active transport requires the hydrolysis of ATP as the energy input necessary to facilitate transport across the cell membrane. To understand whether active transport is involved in the internalization of mono- and di-aryls, *E. coli* cells were grown to mid-log phase, then pelleted and resuspended in media lacking glucose, and challenged to internalize a representative mono-aryl (VA) while energy-starved [21]. VA was chosen because it internalized at a high level in *E.* coli when the cells were not energy-starved (Fig. 5a). Following the 4-h period of exposure to VA accompanied by acute glucose starvation, the levels of VA determined by LC/MS were statistically significantly decreased as compared to non-starved controls (Fig. 7a). To more directly test whether ATP hydrolysis is required for the internalization of these mono- and di-aryls, *E. coli* cells were grown to mid-log phase and then exposed to carbonyl cyanide *m*-chlorophenyl hydrazone (CCCP), a chemical inhibitor of oxidative phosphorylation, during the 4-h internalization period [21]. CCCP is widely used to test for active transport [36–38]. LC/MS showed a decrease in internal VA concentrations following CCCP treatment (Fig. 7b). Complimentary with this, click-labeling followed by single-cell microscopy and analysis showed that *E. coli* cells exposed to CCCP during internalization also showed statistically reduced internal levels of VA (Fig. 7c, d).

## Discussion

Here we have adapted mass spectrometry-based methods for directly quantifying uptake, accumulation, and metabolism in bacteria and fungi, and a microscopy method to provide single-cell statistics and visual observation of the internalized compound. We used these methods to characterize relative intracellular concentrations of four mono-aryls and one di-aryl into four diverse organisms. Prior to this work, only indirect measurements of mono- and di-aryl internalization had been made in microbes [5, 11, 12, 15–20].

*Phanerochaete chrysosporium* has long been known to mineralize lignin [39], but it remained unclear which portion of the depolymerized fragments resulting from the activity of extracellular peroxidases and other oxidative chemistry are internalized and metabolized further intracellularly. Surprisingly, internalization of all five compounds by *P. chrysosporium* occurs for non-lignolytic as well as for lignolytic conditions (Fig. 3). A moderate amount of variation was observed between biological replicates of *P. chrysosporium*, leading to larger error bars than with the other organisms. We believe this is due to the nature of the organism itself. *P. chrysosporium* grows as a network of branched hyphae that form a mycelium, rather than a cell suspension like the other organisms. As such it is challenging to obtain consistent growth and sampling of *P. chrysosporium*. Prior work has shown that growth on microcrystalline cellulose stimulates lignolytic secondary metabolism indicated by upregulation of extracellular and cytoplasmic enzymes relevant to lignolysis after 3 days, whereas growth on glucose results in a non-lignolytic state [40]. While this same publication showed that two transporters are upregulated in the lignolytic state [40], our results indicate that internalization of the five compounds in this study is not mediated by those transporters. The fact that internal concentrations are lower in the lignolytic state may suggest greater metabolism of the compounds in the lignolytic state, but additional studies involving a range of incubation times are needed to confirm this. Metabolism of vanillic acid by *P. chrysosporium* is consistent with a prior report of vanillate hydroxylase activity in cell lysate [41]. The present data provide strong evidence that each of the compounds in this study is internalized by *P. chrysosporium*. We conclude that internalization of lignin-derived monomers and dimers occurs in *P. chrysosporium* in addition to the known extracellular enzymatic pathways that degrade lignin.

The results demonstrating internalization of GGE in *P. chrysosporium* and in *E. lignolyticus* are particularly important (Figs. 1, 3, and 5). Prior to this work, direct evidence for internalization of a model lignin dimer had not been shown. Due to the difficulty of depolymerizing lignin into a stream consisting only of monomers, there is a great need to expand the range of compounds taken up and metabolized to include dimers and potentially higher-order oligomers. The LC/MS results showed greater accumulation of GGE in the lignolytic organism *E. lignolyticus*) compared to the non-lignolytic organisms (*E. coli* and *S. cerevisiae*), suggesting that transport of higher-order aromatic oligomers may require specialized transport systems unique to lignolytic organisms. The lysate concentration of GGE was also much higher in *P. chrysosporium* than for the non-lignolytic organisms, but quantitative comparison cannot be made in that case due to the qualitative nature of cell density in *P. chrysosporium* mycelium growth. The combined observations of internalization and initial catabolism of GGE in *E. lignolyticus* and *P. chrysosporium* along with lack of growth of *E. lignolyticus* on GGE observed by others suggest that sufficient metabolic pathways needed for growth on GGE are lacking and/or that guaiacol may be toxic, as has been reported for many bacterial species [16]. Interestingly, despite clear evidence here of internalization and initial catabolism of GGE in aerobic conditions, *E. lignolyticus* does not grow on mono-aryl compounds as sole carbon source under anaerobic conditions, whether or not guaiacol is present (Kristen DeAngelis, private communication). Based on our results, we can conclude that lack of growth of *E. lignolyticus* on GGE is not due to limited uptake. *E. lignolyticus* may lack pathways for further converting the initial GGE breakdown products. Guiaicol toxicity may also limit growth.

In the non-lignolytic microbes, LC/MS results showed much higher accumulation of vanillic acid in *E. coli* relative to the other compounds and much higher accumulation of *p*-coumaric acid in *S. cerevisiae* relative to the other compounds (Figs. 1 and 5). This suggests the existence of specific, efficient transporters for vanillic acid and *p*-coumaric acid in *E. coli* and *S. cerevisiae*, respectively. Both of these observations are consistent with prior indirect measurements (growth and toxicity studies) for these organisms. Vanillic acid is known to be toxic to *E. coli* [42–44], suggesting that the organism can internalize vanillic acid but is unable to efficiently metabolize or efflux it. *S. cerevisiae* has shown significantly reduced growth rate in the presence of *p*-coumaric acid and only slow catabolic conversion of *p*-coumaric acid over a period of 72 h [27]. Tailoring transport of aromatic compounds in *S. cerevisiae* and other yeasts may minimize toxicity in bioethanol fermentation processes and may also aid the effort to use yeast to convert phenolic compounds into useful chemicals [27, 45].

The observed differences in intracellular concentrations of the five compounds within and between organisms could result from differences in rates of import, efflux, or catabolism. Further studies are necessary to resolve the relative contributions of each of these processes. Combining measurements of target compound internalization with measurements of time-dependent changes in intracellular and extracellular concentrations of metabolites, and detailed studies of any extracellular modification of the target compounds, are exciting directions of future work. However, a few reasonable inferences can be made from the existing data. First, efficient internalization processes must be present if high levels of compounds are detected in cell lysates at short incubation times, such as for vanillic acid uptake by *E. coli* and coumaric acid uptake by *S. cerevisiae.* Second, since the non-lignolytic organisms *E. coli* and *S. cerevisiae* are unlikely to possess efficient catabolic pathways for GGE, we expect that the low levels of GGE detected in cell lysate in those cases are due to very low internalization rates. Third, the significantly greater intracellular GGE concentration in *P. chrysosporium* at 20 min as compared to 4 h, and the presence of the GGE metabolite protocatechuate at 4 h in both *P. chrysosporium* and *E. lignolyticus*, suggests catabolism of this dimer in the lignolytic organisms (Fig. 1, Additional file 8: Figure S8).

While our single-cell microscopy approach is limited to compounds that can be prepared with an alkyne label, it provided a unique look at the cell-to-cell variability. The average value over the cell population obtained by LC/MS provides relative concentrations that can be compared between populations, but the confocal fluorescence microscopy approach provides (1) a complementary tool for answering questions that population measurements cannot—such as the heterogeneity of aryl internalization and (2) verification that the compounds are in fact internalized into the cells, not merely adhered to the cell surface. With the confocal microscopy technique, if the signal arose from compounds bound to the surface of the cells the localization pattern would “ring” the cell periphery. Single-cell analysis for the two compounds revealed large differences in intracellular concentration from cell to cell in a given population (Additional file 5: Figure S5). The observation of heterogeneity of mono-lignol transport at the single-cell level is not necessarily unexpected. Although intracellular transport of mono-lignols has not yet been studied at the single-cell level due to the lack of methodology to do so until now, we and others have observed heterogeneity in other biological processes at the single-cell level, even for organisms from a clonal population [46–48]. This is an active and interesting area of research. Although in our current experiments in *P. chrysosporium*, the signal was brightest in the basidiospores with little to no signal in the fungal hyphae, the technique is capable of interrogating and tracking aromatic compounds in a variety of cell types in future application, including the network of fungal hyphae, with slight changes to the image preprocessing and analysis routines. Future studies could also incorporate live cell click chemistry labeling schemes that have recently been successful for labeling inside living cells [49]. Subsequent sorting of cells with different responses followed by analysis may provide insight into the genetic basis for heterogeneous response.

The fact that both the LC/MS and microscopy methods detected differences in internalization between *E. coli* cells exposed to chemical inhibitors and glucose starvation is an important finding of this study (Fig. 7). It indicates that internalization of VA by *E. coli* occurs via active transport, and the same may hold true for the other compounds and other organisms. Active transport is one possible mechanism by which these mono- and di-aryls can be internalized by bacteria and fungi, but passive transport mechanisms may also contribute. The energy dependence of internalization observed for VA in *E. coli* was immediate as cells were not pretreated when challenged with glucose starvation or oxidative phosphorylation during the 4-h internalization time course. These data strongly suggest that transporters are involved in the internalization of the representative mono-aryls by *E. coli*. Studies of engineered organisms will yield more information about the role specific transporters play in internalization of lignin-derived compounds. When applied at multiple incubation time points, as shown here for vanillic acid accumulation in *P. chrysosporium* versus *S cerevisiae* (Fig. 4), relative rates of internalization and metabolism can be determined.

## Conclusions

We developed two methods for measuring internal mono- and di-aryl concentrations in fungi and bacteria, one at the population level and one at the single-cell level. We have demonstrated the utility of these methods in detecting differences in internalization among a small set of compounds for individual organisms, differences in internalization between lignolytic or non-lignolytic organisms, as well as detecting internalization differences in response to chemical inhibitors or glucose starvation. We observed broad variation in intracellular concentration within populations, and this may have important consequences for the efficiency and productivity of an industrial process for bioconversion. This work lays the groundwork for understanding and quantifying internalization of extracellular lignin degradation products and for determining how pathways can be engineered to most efficiently transport and degrade mono- and di-aryls. Subsequent application of this toolset will be useful in identifying and characterizing specific transporters for lignin-derived mono- and di-aryl compounds.

## Materials and methods

### Culture conditions

#### *Phanerochaete chrysosporium*—(Burdsall ATCC^®^34541™)

One milliliter of *Phanerochaete chrysosporium* stock was administered to a Potato Dextrose Agar (PDA) plate and evenly spread around the plate using a plastic spreader. These plates were stored in a 37 °C humidified oven and allowed to grow for 48 h minimum. *P. chrysosporium* plates were maintained weekly by cutting a small (~ 1 cm) sample out of the agar of the current plate and placing the sample on fresh PDA plates with the spore-covered side face-down into the fresh agar. Cells were seeded into liquid media from these PDA plates 5 days prior to uptake trial. Two milliliters of fungal media (Kirk’s mineral salts and trace elements solution, prepared as in [50]) supplemented with either 2 mg/mL microcrystalline cellulose (Avicel^®^ PH-101 from Sigma-Aldrich 11365-1 KG) for lignolytic growth conditions or 10 mg/mL glucose for non-lignolytic growth) was inoculated with 100 μL of spore suspension drawn from PDA plate. Plates were sealed to prevent media evaporation and placed in a humidified 37 °C oven for 5 days to allow mycelia to form.

#### *Saccharomyces cerevisiae*—(INVSc1 Invitrogen™; Cat. No. C810-00)

Single colonies grown on PDA (potato dextrose agar) plates were picked and grown overnight at 30 °C in 5 mL of YPD (1% Yeast extract, 2% Peptone, 2% Dextrose). A 5 mL starter culture was used to inoculate 50 mL of YPD to an OD600 of 0.1. These were grown for approximately 14 h at 30 °C until they reached an OD600 of 1. Cultures were then split into 5 mL subcultures for aryl addition.

#### *Enterobacter lignolyticus*—(SCF1 from DSMZ culture collection)

Single colonies grown on nutrient broth (Difco™ BD 234000) agar plates were picked and grown overnight at 28 °C in 5 mL of nutrient broth. The 5 mL starter culture was then used to inoculate 50 mL of nutrient broth to a starting OD600 of 0.1. Cultures were grown at 28 °C with shaking until they reached OD600 0.4 (approximately 4 h). Cultures were then split into 5 mL subcultures for aryl addition. A minimal growth media was used in place of nutrient broth to look for GGE metabolites, recipe from Kristin DeAngelis lab.

#### *Escherichia coli*—[(Migula) Castellani and Chalmers (ATCC^®^ 700926™)]

Single colonies grown on Luria broth base (Invitrogen 12795-027) agar plates were picked and grown overnight at 37 °C in 10 mL of M9 media (Barrick Lab recipe + 1 µg/mL thiamine, 40 µg/mL l-glutamine and l-arginine, and 0.4% glucose). The 10 mL starter culture was then used to inoculate 50 mL of M9 media to a starting OD600 of 0.1. Cultures were grown at 37 °C with shaking until they reached OD600 0.4 (approximately 16 h). Cultures were then split into 5 mL subcultures for aryl addition.

### Incubation with mono- or di-aryl substrates

Mono-aryls tested were vanillic acid (Sigma-Aldrich 94770), 4-hydroxybenzoic acid (Sigma-Aldrich H20059), *p*-coumaric acid (Sigma-Aldrich C9008), and syringic acid (Sigma-Aldrich S6881). Di-aryl tested was guaiacylglycerol-beta-guaiacyl ether (Sigma-Aldrich CDS013307). *E. coli* and *E. lignolyticus* were grown to OD600 0.4, and mono- and di-aryl DMSO solutions were diluted to 4 mM in the growth media (see culture conditions for media and specific growth conditions). Cultures were incubated with mono- and di-aryls for 4 h at 37 °C (*E. coli*) or 28 °C (*E. lignolyticus*) with shaking. *S. cerevisiae* cultures were grown to an OD600 of 1.0, and mono- and di-aryl DMSO solutions were diluted to 4 mM in the growth media (see culture conditions for media and specific growth conditions). Cultures were incubated with aryls for 4 h at 30 °C with shaking. For microscopy samples, 400 μL of OD600 0.4 (bacteria) or OD600 1.0 (yeast) cultures was transferred to each well of poly-l-lysine (Sigma-Aldrich A-005-C) coated glass imaging chambers (ThermoFisher Scientific 155409) and shaken at appropriate temperatures for 4 h in the presence of 4 mM alkyne-modified aryls in the media (see “Fluorescence microscopy” section). *P. chrysosporium* media was changed to Kirk’s mineral media containing 4 mM of each mono- and di-aryl. Incubation for 20 min or 4 h was done in a humidified oven at 37 °C without shaking. *P. chrysosporium* microscopy samples were exposed to alkyne-modified aryls in a 6-well plate format in 2 mL of media for 4 h at 37 °C. For all organisms, following the incubation with aryls, cells were pelleted at 4500 rpm for 10 min. Supernatants were retained for HPLC analysis to determine remaining aryl concentration, and cell pellets were washed 3× with DPBS and then snap-frozen in liquid nitrogen.

### Carbonyl cyanide 3-chlorophenylhydrazone (CCCP) and glucose starvation

*Escherichia coli* were grown to OD600 0.4 in M9 media containing 0.4% glucose as above. For CCCP, once cultures reached OD600 0.4, CCCP was added to the cultures at a concentration of 50 µM for 5 min. Then the mono-aryl was added and incubated as above. The CCCP was present throughout the 4-h incubation. Cells were pelleted, washed, and prepared for TQD-MS analysis or washed and fixed to prepare for imaging. For glucose starvation, once cultures reached OD600 of 0.4, cultures were pelleted at 4500 rpm for 10 min. M9 media was removed, and cells were washed once with PBS, spun down again and then resuspended in M9 media containing no glucose. The mono-aryl was then added, and incubation and harvesting for TQD-MS occurred as described.

### Manganese peroxidase activity assay

Manganese peroxidase activity of *P. chrysosporium* grown in Kirk’s mineral media containing either 2 mg/mL microcrystalline cellulose or 10 mg/mL glucose was assayed by concentrating secreted protein in the media following 5 days of growth using a 10 K cutoff filter, adding 20 μL of the retentrate to 980 μL of 50 mM sodium malonate buffer pH 4.5 containing 0.5 mM MnCl_2_ and 0.2 mM H_2_O_2_ and measuring absorbance at 270 nm using a NanoDrop™ 2000 spectrophotometer at *T* = 1, 2, 5, and 10 min.

### Mass spectrometry

#### Cell lysis and sample purification

Cell pellets were thawed on ice for 1 h. One milliliter of 70% HPLC-grade ethanol (Sigma-Aldrich 459828) was added to pellets on ice. Cells were resuspended then transferred to lysing matrix tubes. Lysing matrix C (Fisher Scientific MP116912050) was used for fungal samples, and lysing matrix B (Fisher Scientific MP116911050) was used for bacterial samples. Cells were agitated at 2000 rpm using a Cell Disrupter Genie at 4 °C for 2 h. Lysates were then placed over a 3 kDa filter (Sartorius VS0192) and spun at 15,000×*g* for 2 h at 4 °C to clarify the lysates.

#### Instrument details, data collection parameters, and analysis—QToF-MS

LC/MS on cell lysates for Figs. 1, 3, and 5 and the supporting data in Additional file 1: Figure S1, Additional file 2: Figure S2, Additional file 8: Figure S8, Additional file 9: Figure S9, and Additional file 11: Figure S11 was carried out using a Waters ACQUITY Ultra Performance LC system linked to Xevo-G2-XS-QToF mass spectrometer (Waters, Milford, MA, USA). MassLynx software (version 4.0, Waters, Milford, MA, USA) was used to control the instrument and for data acquisition and processing. Ten microliter sample solutions were injected into a F5 (pentafluorophenyl) reverse-phase column (2.6 µM, 100 Ǻ, 100 × 2.1 mm, Phenomenex, Torrance, CA, USA). The column was maintained at 40 °C. The mobile phase consisted of the following 20 min sequence of linear gradients: from 0.1% formic acid + water to 0.1% formic acid + 95% acetonitrile. At the end of this sequence the column was equilibrated under initial conditions for 2 min. The pressure ranged from 3000 to 6000 psi during the chromatographic run. The effluent was introduced into an electrospray ionization (ESI) source operating in negative mode. The ESI settings were an ionization voltage of 2.5 kV, source temperature of 120 °C, sampling cone at 40.0 V, extraction cone at 4.0 V, acquisition mass range of 50–700 Da, scan time of 0.5 s, detector at 2000 V, nitrogen was used as desolvation gas (600 L/h), and data format was set to continuum.

Calibration curves of 0–0.1 mM for each aryl were prepared in methanol to determine the linear range for our instrument (Additional file 11: Figure S11). Based on these curves, 0.005 mM was selected from the linear range as an optimal concentration for a lysate spike. In order to calculate relative internalized lysate concentrations based on ion counts, spiked 0.005 mM standards of each aryl were prepared in cell lysate from each organism. Freshly spiked standards were run for all aryls for each internalization experiment. Intracellular concentrations were calculated based on total ion counts over peak width. GGE fragmentation occurred due to the presence of salt from the cell washes. Therefore, ion counts from peaks at 319 and 271 were added for all GGE data. Lysates from each organism with DMSO vehicle controls were also run to identify and subtract any vehicle-associated peaks. Each experimental condition was performed in triplicate and plotted as mean with standard deviation using GraphPad (GraphPad Software, Inc., La Jolla, CA, USA).

#### Instrument details, data collection parameters, and analysis—ASAP-MS

Atmospheric solid analysis probe–mass spectrometry (ASAP-MS) experiments were performed early on in our studies for the results shown in Fig. 4 and Additional file 7: Figure S6, but we chose to perform most of our measurements using electrospray ionization due to much greater sensitivity and more accurate quantification. The ASAP-MS measurements were performed on a Synapt G2-ToF high-definition mass spectrometer (Waters Corporation, Milford, MA, USA). MassLynx software (version 4.0, Waters, Milford, MA, USA) was used to control the instrument and for data acquisition and processing. Cell lysates were deposited on the end of ASAP glass capillary probe by allowing 10 µL of sample solutions to air dry on the probe. The probe temperature was initially set at 100 °C for 1 min, increased to 150 °C for another min, and then increased in 50 °C increments every 30 s to 600 °C. ToF-MS was set at positive ionization mode with source temperature of 120 °C, sampling cone at 48.0 V, extraction cone at 4.0 V, corona current at 8 μA, desolvation gas at 1000 L/h, acquisition mass range of *m/z* 50–700, and scan time of 0.5 s. Mass spectra collected at  > 350 °C were used as resulting spectrum, and < 350 °C were used as background. Vanillic acid ion counts were calculated by subtracting background from resulting spectrum. The counts for vanillic acid (*m/z* = 168.02) and 6C13 vanillic acid (*m/z* = 174.03) were normalized by rationing to the counts at *m/z* = 219.02 (*P. chrysosporium*) and *m/z* = 185.02 (*S. cerevisiae*). The results for the 1-h time point were normalized to a value of 1 to correct for daily variations in instrument performance.

#### Instrument details, data collection parameters, and analysis—TQD-MS

Ultrahigh-performance liquid chromatograph–mass spectrometry was performed on a tandem quadrupole mass spectrometer (TQD-MS) (Waters Corporation, Milford, MA, USA) for the CCCP and starvation experiments shown in Fig. 7. MassLynx software (version 4.1, Waters, Milford, MA, USA) was used to control the instrument and for data acquisition and processing. The mass spectrometer was first tuned to each compound by direct infusion of the solutions of each analyte at 50 parts per million (ppm) in ethanol solvent. Cone voltage and collision energy were optimized to each compound. Three microliters of sample solutions was injected into a Waters ACQUITY BEH C18 reverse-phase column (1.7 µM 2.1 × 100 mm). The column is maintained at 40 °C. The mobile phase (A: 0.1% formic acid + water; B: 0.1% formic acid + acetonitrile) consisted of the following 15-min gradient sequence: initial: 95% A/5% B; then 95% A/5% B to 5% A/95% B in 10 min, hold 5% A/95% B for 2 min; equilibrate to initial conditions for 3 min. The general ESI settings were: an ionization voltage of 2.0 kV, source temperature of 150 °C, sampling cone at 40.0 V, desolvation temperature at 350 °C, desolvation gas flow at 850 L/h. The optimized conditions for each compound are listed as: 1. 4-hydroxybenzoic acid (4-HBA): optimized cone voltage at 30 V, mass transition measured: (1) 136.9343 *m/z* → 64.9896 *m/z* at 28 V; (2) 136.9343 *m/z* → 93.3351 *m/z* at 24 V at elution time of 1.65 min. 2. *p*-coumaric acid (CA): optimized cone voltage at 30 V, mass transition measured: (1) 162.9443 *m/z* → 93.0006 *m/z* at 28 V; (2) 162.9443 *m/z* → 93.5162 *m/z* at 24 V; (3) 162.9443 *m/z* → 119.5234 *m/z* at 28 V at elution time of 1.83 min. 3. vanillic acid (VA), optimized cone voltage at 32 V, mass transition measured: (1) 166.8804 *m/z* → 90.9167 *m/z* at 18 V; (2) 166.8804 *m/z* → 107.9384 *m/z* at 18 V; (3) 166.8804 *m/z* → 151.9479 *m/z* at 14 V at elution time of 1.71 min. 4. guaiacylglycerol-beta-guaiacyl ether (GGE), optimized cone voltage at 28 V, mass transition measured: (1) 319.0881 *m/z* → 149.1134 *m/z* at 32 V; (2) 319.0881 *m/z* → 256.0845 *m/z* at 22 V; (3) 319.0881 *m/z* → 271.0973 *m/z* at 12 V at elution time of 2.04 min. Calibration verification samples were injected throughout sample analysis to monitor the accuracy and precision of the calibration during sample analysis. A lysate cell matrix blank was also injected throughout sample analysis to ensure no sample was contaminated with targeted analytes during sample preparation or analysis.

### High performance liquid chromatography (HPLC)

#### Sample preparation

PBS supernatants were thawed on ice and were filtered over a 3-kDa column spin filter (Sartorius VS0192) prior to injection onto the column.

#### Instrument details, data collection parameters

Analysis of the concentration of mono- and di-aryl remaining in PBS following the experiment was determined using high-performance liquid chromatography (HPLC) using an Agilent 1260 Infinity HPLC unit equipped with a Phenomenex Kinetex 00F-4601-E0 C18 column. The mobile phase ramped from 95% water (18 mΩ), 5% acetonitrile (Sigma-Aldrich 34851) to 30% water, 70% acetonitrile over the course of 8 min. A 3-min column purge at 70% acetonitrile, 30% water was held followed by return to the starting 5% acetonitrile, 95% water. The flow rate was 1 mL/min. Wavelength of detection varied between compounds to provide maximal detection capability, using 251 nm, 246 nm, and 277 nm for vanillic acid, 4-hydroxybenzoic acid, and guaiacylglycerol-beta-guaiacyl ether, respectively.

#### Calibration curves/quantification

Calibration curves of 1, 2, and 4 mM spiked standards were prepared in PBS. Four millimolars of spiked standards in PBS were used to quantify relative concentration of aryl remaining in the supernatant for experimental samples.

### Click chemistry-enabled single-cell microscopy

#### Synthesis of alkyne labeled substrates

All operations were carried out under argon atmosphere using standard Schlenk techniques, unless otherwise noted. All reagents and anhydrous solvents are commercially available and were used as received.

#### Synthesis of fHBA

The alkyne-functionalized 4-hydroxybenzoic acid (fHBA) was synthesized by a similar procedure to that described in Zhu et al. [51] as detailed below.

##### Synthesis of fHBA-CH_3_ (methyl-4-(2-propynyloxy)benzoate)

Methyl-4-hydroxybenzoate (5.01 g, 32.9 mmol) and potassium carbonate (10.25 g, 74.2 mmol) were combined in a two-necked 100-mL round-bottom flask fitted with a reflux condenser. The assembly was purge/vac’d with argon three times, and then 20 mL anhydrous acetonitrile added via syringe. After stirring briefly 4.5 mL (41.4 mmol) of propargyl bromide (80% in toluene) were added via syringe. The reaction mixture was refluxed under Ar overnight. The reaction was cooled to room temperature and quenched with approximately 30 mL H_2_O, added in small aliquots. The reaction mixture was extracted with 3 × 15 mL ethyl acetate. The combined organic portions were washed with brine and dried over magnesium sulfate. The solution was filtered and rotovapped to yield a brownish oil, from which precipitated colorless crystals after several hours. The oil was decanted from the crystals, which were washed with hexanes to yield pure methyl-4-(2-propynyloxy)benzoate in 66% yield. ^1^H NMR (500 MHz, CDCl_3_): *δ* 8.00 (d, *J* = 8.2 Hz, 2H, CH_Ar_), 6.99 (d, *J* = 8.1 Hz, 2H, CH_Ar_), 4.74 (s, 2H, CH_2_), 3.88 (s, 3H, CH_3_), 2.55 (s, 1H, C≡CH).

##### Synthesis of fHBA (4-(2-propynyloxy)benzoic acid)

fHBA-CH_3_ (2.00 g, 10.6 mmol) was combined with anhydrous LiOH (2 g, 85 mmol) in a two-necked round-bottom flask fitted with a reflux condenser. The assembly was purge/vac’d with Ar three times. Meanwhile, 15 mL of ethanol and 2 mL of water were combined and degassed and then added to the reaction flask via syringe. The mixture was refluxed under Ar overnight and then cooled to room temperature under Ar. The reaction mixture was transferred to a large round-bottom flask and 100 mL of methanol added. The mixture was rotovapped at 40 °C until ethanol/methanol was removed, leaving a damp powder. After rotovapping, 30 mL of 1 M HCl were added and the reaction mixture stirred until all the excess LiOH had dissolved. Additional 1 M HCl was added until the pH was adjusted to < 2. Copious white precipitate formed on acidification. The precipitate was filtered and washed with water forming a thick white paste. The precipitate was dried at room temperature overnight. Hot recrystallization from ethanol (cool to RT then recrystallize at 4 °C) yielded very fine iridescent plates of the product in 76% yield. ^1^H NMR (500 MHz, d_6_-DMSO): *δ* 12.69 (s, 1H, COOH), 7.91 (d(m), *J*_d_= 8.9 Hz, 2H, CH_Ar_), 7.06 (d(m), *J*_d_ = 9.0 Hz, 2H, CH_Ar_), 4.89 (d, *J* = 2.4 Hz, 2H, CH_2_), 3.62 (t, *J* = 2.4 Hz, 1H, C≡CH).

#### Synthesis of alkyne-functionalized VA (fVA)

The alkyne-functionalized vanillic acid analog (fVA) was synthesized by a method similar to that reported by Bukowski et al. for the synthesis of a mono-functionalized caffeyl alcohol analog [52].

#### Synthesis of alkyne-functionalized VA (fVA)

The alkyne-functionalized vanillic acid analog (fVA) was synthesized by a method similar to that reported by Bukowski et al. for the synthesis of a mono-functionalized caffeyl alcohol analog [52].

##### Synthesis of fVA-CH_3_ (methyl 3,4-bis(2-propynyloxy)benzoate)

Sodium hydride (585 mg, 24 mol) was placed into a two-necked round-bottom flask and purge/vac ×3 with argon. Anhydrous DMSO, 125 mL, was transferred in via cannula. The reaction was cooled until the DMSO formed a slush, and then methyl 3,4-dihydroxybenzoate (1.02 g, 6.1 mmol) was added against a positive flow of Ar. The reaction was allowed to warm slowly to room temperature and stirred at room temperature for 30 min. The solution was then cooled down until the DMSO formed a slush and 0.67 mL (6.2 mmol) of propargyl bromide (80% in toluene) was added. The solution begins to turn brown ~ 1 min after addition. The solution is allowed to gradually warm to room temperature and is then stirred overnight at room temperature. The reaction mixture was quenched with 10 mL of methanol added in small aliquots. To the quenched reaction mixture was added 30 mL of ethyl acetate (monophasic) followed by 100 mL of water, which initiates phase separation. Separate the organic phase and wash the aqueous phase with 2 × 15 mL ethyl acetate. Combine the organic fractions and wash with 1 × 20 mL each of 1 M HCl, water, and brine. Dry with magnesium sulfate and filter. Rotovap to yield a brown oil which is a mix of the mono-functionalized (methyl 3-(2-propynyl)benzoate) and di-functionalized (methyl 3,4-(2-propynyl)benzoate) products. Purify by column chromatography with 65:35 hexanes/ether yielding pure di-functionalized (early fractions) and mono-functionalized (later fractions) product. A second column with the same solvent system was run to purify the mixed fractions. The combined yield of pure di-functionalized fVA-CH_3_ was 20% vs benzoate starting material (294.1 mg, 1.2 mmol). (Note that reaction stoichiometry was targeted toward the mono-functionalized product. Yield of di-functionalized fVA-CH3 could be optimized by adjusting the amount of propargyl bromide). ^1^H NMR (500 MHz, d_6_-acetone): *δ* 7.69 (m, 1H, CH_Ar_), 7.67 (d, *J* = 2.0 Hz, 1H, CH_Ar_), 7.20 (d, *J* = 8.2 Hz, 1H, CH_Ar_), 4.91 (d, *J* = 2.4 Hz, 2H, CH_2_), 4.87 (d, *J* = 2.4 Hz, 2H, CH_2_), 3.84 (s, 3H, OCH_3_), 3.13 (t, *J* = 2.4 Hz, 1H, C≡CH), 3.10 (t, *J* = 2.4 Hz, 1H, C≡CH).

##### Synthesis of alkyne-functionalized VA (fVA) (3,4-bis(2-propynyloxy)benzoic acid)

fVA-CH_3_ **(**di-functionalized) (294 mg, 1.3 mmol) was placed in a two-necked round-bottom flask fitted with a reflux condenser. The assembly was purge/vac’d ×3 with argon. Meanwhile, 20 mL of ethanol and 5 mL of water were combined and degassed, and then 5 mL of the mixture was added to the reaction flask via syringe. LiOH (185 mg, 7.7 mmol) was added against a positive flow of argon, and the reaction mixture was refluxed under argon for 6 h. The reaction mixture was transferred to a 50-mL round-bottom flask with 20 mL of MeOH and rotovapped to yield a white slurry. 1 M HCl was added until the solution cleared, and then copious amounts of white precipitate formed on further acidification (final pH < 3). The white precipitate is filtered and washed thoroughly with water and then dried overnight. The product is recrystallized from hot ethanol (cool to RT then recrystallize at 4 °C) to yield the product in 72% yield (215 mg, 0.93 mmol). ^1^H NMR (500 MHz, d_6_-DMSO): *δ* 12.78 (s, 1H, COOH), 7.60 (m, 2H, CH_Ar_), 7.14 (d, *J* = 8.4 Hz, 1H, CH_Ar_), 4.90 (d, *J* = 2.4 Hz, 2H, CH_2_), 4.86 (d, *J* = 2.4 Hz, 2H, CH2), 3.63 (t, *J* = 2.4 Hz, 1H, C≡CH), 3.61 (t, *J* = 2.4 Hz, 1H, C≡CH). *Note:* The mono-functionalized vanillic acid analog could not be synthesized due to cyclization to a 1,4–benzodioxine during deprotection of the acid (see Additional file 3: Figure S3).

#### Incubation with alkyne-functionalized aryls

See “Incubation with mono- or di-aryl substrates.”

#### Click chemistry labeling

After cells were incubated during log-phase growth in the presence of the alkyne-functionalized aryls for 4 h, cells were fixed with 4% paraformaldehyde in Nunc™ Lab-Tek™ chambered coverglasses (for *E. coli*, *E. lignolyticus*, and *S. cerevisiae*) or in solution (*P. chrysosporium*). Cells were then permeabilized in a 0.1% Triton-X PBS solution for 15 min. Following the fixation and permeabilization reactions, the alkyne-functionalized aryls were fluorescently labeled using the Click-iT™ Plus EdU AlexaFluor™ 647 Imaging Kit (Invitrogen C10640). Cells were stained with DAPI at 1 μg/mL and were then mounted onto slides or in the imaging chambers using ProLong™ Gold Antifade Mountant (ThermoFisher Scientific P36930).

#### Fluorescence microscopy

Fixed cell microscopy was done on an Olympus IX-71 fluorescence microscope equipped with a Q Imaging^®^ Rolera EM-C^2^ ™ EMCCD camera for all results except Fig. 7. Microscopy was performed using an Olympus PlanApo N 60×/1.42 oil immersion objective. A white light Prior Lumen 200PRO excitation source was used, and filters used were Semrock DAPI-1160A-OMF-ZERO (EX 387/11, EM 447/60, DI 409) and Semrock Cy3/Cy5-2X-A-OMF (Pinkel, EM1 577/24, EM2 690/50, DI 560/659-Di01) with excitation filter Semrock 628/40 (Cy5/DRAQ5). SlideBook 6.0.4 × 64 from Intelligent Imaging Innovations, Inc., was used as the image acquisition software. Wide-field microscopy was performed on each of three biological replicates for each experimental condition. For each condition within a replicate, 5–10 fields of view were collected at 60× magnification. This yielded ≥ 300 cells per organism per condition for single-cell analysis with the exception of *P. chrysosporium*. For data shown in Fig. 7, fixed cell microscopy was performed using a Leica DMi8 DLS confocal fluorescence microscope equipped with a Leica HC PL APO CS2 63×/1.40 oil immersion objective. Multiple laser diode excitation sources were used (405 nm and 638 nm) with Leica SP8 LIAchroic low incident angle dichroic beam splitters and PMT detectors. Images were acquired using LAS X Version 3.5.2.18963 by Leica Microsystems ©2018 in sequential scanning mode using a line averaging of 4 with the following settings—3% 405 nm excitation, detecting 415–485, 600 V gain PMT; 2% 638 nm excitation, detecting 650–690 nm, 500 V gain PMT.

#### Image analysis—single-cell quantification

Sixteen-bit tiff images for all organisms and conditions were analyzed to individually segment cells in ImageJ (NIH, Bethesda, MD). The DAPI channel for each field was used to generate a mask for the cell positions. For the *S. cerevisiae* images, the DAPI channel was preprocessed using a maximum intensity filter with a 7-pixel sliding window to smooth out irregularities in DAPI labeling and permit automated cell segmentation. A watershed algorithm was then applied to the image to assist in splitting touching cells. The coordinates and outlines of the individually segmented cells were determined from the DAPI image using Analyze > Analyze Particles and then were applied to the AlexaFluor™ 647 channel 16-bit tiff image to quantify a set of parameters in each cell including area, integrated density, min, mean, and max gray value, and perimeter. Prior to quantification, the AlexaFluor™ 647 images were background corrected using a rolling ball background correction with no smoothing. A relevant radius was selected for each organism based on their size (*E. coli*, 10 pixels; *E. lignolyticus*, 20 pixels; *S. cerevisiae*, 20 pixels; and *P. chrysosporium*, 150 pixels). Results were then filtered by the measured area to exclude objects that were either too small or too large to represent individual cells from the organism in the images. Three regions outside of segmented cell regions were measured in each AlexaFluor™ 647 image to determine background signal intensity. To assess the DMSO images for positive cells, the intensity of this AlexaFluor™ 647 background signal was averaged over all DMSO images in a particular organism. A conservative threshold of a mean intensity > 7× the standard deviation of the average background intensity was chosen to mark a cell as “positive” in the DMSO images. These cells are not positive in the sense that they contain the modified compounds, as they were not exposed to them, but rather the positive signal represents a combination of inherent native autofluorescence and possibly presence of alkyne bonds in the native organism that get labeled. It should be noted that except for *P. chrysosporium* the autofluorescence levels in the DMSO images were low. To quantify the percent AlexaFluor™ 647 positive cells from the images of cells exposed to the VA analog and 4-HBA analog, thresholds were set that represented approximately 3× the autofluorescence level in the DMSO images for that organism (*E. coli* & *E. lignolyticus*, 300 cts for VA analog and 100 cts for 4-HBA analog; *S. cerevisiae*, 1000 cts for VA analog and 800 cts for 4-HBA analog; and *P. chrysosporium*, 1600 cts for VA analog and 600 cts for 4-HBA analog). Thresholds are slightly higher for the VA analog due to VA alkyne-functionalized mono-lignol having 2 click sites. Images with this compound exhibited non-specific background and signal levels about 1.5- to 2-fold higher than the mono-functionalized 4-HBA analog. All thresholds were verified by comparison with manual counts on at least half the images for each organism and condition. For image panels, AlexaFluor™ 647 intensities are scaled equally within organisms so AlexaFluor™ 647 intensities can be compared within panels. DAPI scaling varies within panels for optimal display purposes so should not be quantitatively compared. For the single-cell average intensity plots in Additional file 5: Figure S5, the mean intensities for individual intensities are presented raw and do not compensate for the VA alkyne-functionalized mono-lignol having two click sites. Any increase above a twofold difference shows enhanced internalization of the VA alkyne-functionalized mono-lignol relative to the 4-HBA alkyne-functionalized mono-lignol. The single-cell analysis for the CCCP experiment is displayed without thresholding for autofluorescence. Sixteen-bit tiff images were analyzed to individually segment the *E. coli* cells in ImageJ (NIH, Bethesda, MD). The DAPI channel for each field was used to generate a mask for the cell positions, and the single-cell integrated intensities of the AlexaFluor™ 647 channel were measured using that DAPI mask. Four regions outside of segmented cell regions were measured in each AlexaFluor™ 647 image to determine background signal intensity. Cells with mean intensities 3× above the standard deviation of that average background were included in the single-cell analysis, and the integrated intensities of those cells are graphed in Fig. 7.

## Additional files

**Additional file 1: Figure S1.** A. Representative mass spectra (QToF-MS) for each uptake compound (4-HBA, VA, SA, CA, and GGE) in *P. chrysosporium* lysates. DMSO vehicle control mass spectra are shown at the retention time for each compound of interest for peak comparison. Red arrows mark the integrated peak for results in B (4 h). B. Ion counts for each compounds in *P. chrysosporium* cell lysates from 3 biological replicates. Error bars are standard deviation. C. Ion counts for spiked *P. chrysosporium* lysate with indicated compounds at a concentration of 0.005 or 0.0025 mM. Spikes were used to calculate the relative lysate concentrations in Fig. 1 from ion counts in Fig. S1B. D. Representative mass spectra for protocatechuate (GGE metabolite) in *P. chrysosporium* lysates at 4 h. DMSO vehicle control mass spectra shown at the same retention time for peak comparison. Black arrows mark the integrated metabolite peak for Fig. 1c.

**Additional file 2: Figure S2.** A. Representative mass spectra (QToF-MS) for each uptake compound (4-HBA, VA, SA, CA, and GGE) in *S. cerevisiae* lysates. Integrated peak for internalization experiments is marked with a red arrow. DMSO vehicle control mass spectra are shown at the retention time for each compound of interest for peak comparison. B. Ion counts for each compounds in *S. cerevisiae* cell lysates from 3 biological replicates. C. on counts for spiked *S. cerevisiae* lysate with indicated compounds at a concentration of 0.005 mM. Spikes were used to calculate the relative intracellular concentration in Fig. 1 from ion counts in Fig. S2C.

**Additional file 3: Figure S3.** A monofunctionalized vanillic acid analog, where the methoxy group in vanillic acid was replaced by the propynyloxy group and the p-hydroxy remained unfunctionalized, (Fig. S3, box) would perhaps be a better vanillic acid mimic by preserving the hydrogen bonding of the p-hydroxy.

**Additional file 4: Figure S4.** Table summarizing single cell fluorescence microscopy results.

**Additional file 5: Figure S5.** Single cell AlexaFluor™ 647 intensities of A. *S. cerevisiae* B. *P. chrysosporium* V. *E. coli*, and D. *E. lignolyticus*. Thin red line is the mean and red whiskers mark the standard deviation. Intensities shown are from the cells deemed positive in three biological replicates based on stringent thresholds to minimize the moderate levels of autofluorescence (see “Materials and Methods” for detailed description). Mean intensity data is presented raw and does not compensate for the vanillic acid analog being difunctionalized.

**Additional file 6: Figure S7.** Manganese peroxidase assay measuring absorbance at 270 nm of concentrated Kirk’s mineral media following 5 days of *P*. *chrysosporium* growth. Media contained either 2 mg/mL microcrystalline cellulose (blue line) or 10 mg/mL glucose (red line) during the 5 day growth period. N = 3 biological replicates (each point is average of 3 media measurements).

**Additional file 7: Figure S6.** A. ASAP-MS representative spectra of *S. cerevisiae* lysates incubated with vanillic acid for 1, 2, 3, or 17 h. Blue arrow indicates integrated peak of interest for results displayed in Fig. 4c. B. ASAP-MS representative chromatogram of *P. chrysosporium* lysates incubated with vanillic acid for 1, 2, 4, or 24 h. Orange arrow indicates integrated peak of interest for results displayed in Fig. 4b. C. ASAP-MS representative chromatogram of *P. chrysosporium* lysates incubated with 13C labeled vanillic acid for 1, 2, 4, or 24 h. Red arrow indicates integrated peak of interest for results displayed in Fig. 4b. D. ASAP-MS representative chromatogram of *P. chrysosporium* lysates incubated with vanillic acid for 5, 15, and 60 min. Orange arrow indicates integrated peak of interest for results displayed in Fig. 4b.

**Additional file 8: Figure S8.** A. Representative mass spectra (QToF-MS) for each uptake compound (4-HBA, VA, SA, CA, and GGE) in *E*. *coli* lysates. Integrated peak for internalization experiments is marked with a red arrow. DMSO vehicle control mass spectra are shown at the retention time for each compound of interest for peak comparison. *E. lignolyticus* spectra look similar (data not shown). B. ion counts for each compound in *E*. *coli* vs. *E. lignolyticus* cell lysates from 3 biological replicates. Error bars are standard deviation. C. Ion counts for spiked *E*. *coli* or *E. lignolyticus lysate* with indicated compounds at a concentration of 0.005 mM. Spikes were used to calculate the relative intracellular concentrations in Fig. 5.

**Additional file 9: Figure S9.** A. Representative mass spectra for GGE metabolites (guiacol, protocatechuate, and 2-(2-methoxyphenoxy)-1,3-propanediol) from *E. lignolyticus* lysates grown in minimal media and incubated with DMSO or GGE for 4 h. Integrated peak for each (from left to right in order listed above) is marked with a red arrow. B. Ion counts for integrated peaks indicated with arrows in A. Gray bars are ion counts of each compound for DMSO lysate, and black bars are ion counts of each compound for GGE lysates. 1 biological replicates is shown. C. Representative mass spectra for GGE metabolics (guiacol, protocatechuate, and 2-(2-methoxyphenoxy)-1,3-propanediol) from *E. lignolyticus* lysates grown in nutrient broth and incubated with DMSO or GGE for 4 h. Integrated peak for protocatechuate is marked with a red arrows. D. Average ion counts for integrated protocatechuate peak indicated with arrows in C. 3 biological replicates are shown, error bars are standard deviation. Gray bars are ions counts for DMSO lysate and black bars are ion counts for the GGE lysates.

**Additional file 10: Figure S10.** Area (pX^2^) of *E. lignolyticus* cells following internalization of 4-HBA or VA for 4 h. Cells areas were measures using DAPI signal. ****p < 0.0001 by Kruskal–Wallis test. Red bars mark the average and whiskers are the standard deviation. N = 3 biological replicates. B. Aspect ratio measurements of *E. lignolyticus* cells following internalization of 4-HBA or VA for 4 h. Cells aspect ratios were measured using the longest and shortest axis using DAPI. signal. ****p < 0.0001 by Kruskal-Wallis test. Red bars mark the average and whiskers are the standard deviation. N = 3 biological replicates.

**Additional file 11: Figure S11.** A. LC/MS standard curve of 4-HBA prepared in methanol at 0.001, 0.005, 0.01, 0.05, 0.1 mM. b. LC/MS standard curve of VA prepared in methanol at 0.001, 0.005, 0.01, 0.05, and 0.1 mM. C. LC/MS standard curve of CA prepared in methanol at 0.001, 0.005, 0.01, 0.05, 0.1, 0.25, 0.5, 0.8, and 1 mM. D. LC/MS standard curve of GGE prepared in methanol at 0.005, 0.01, 0.05, and 0.1 mM. E. LC/MS standard curve of SA prepared in 10% methanol, 90% PBS at 0.01, 0.02, and 0.05 mM.

## Data Availability

All datasets used and/or analyzed during this study are available from the corresponding author on reasonable request.

## Abbreviations

GGE: guaiagylglycerol beta-guaiacyl ether
4-HBA: 4-hydroxybenzoic acid
VA: vanillic acid
CA: *p*-coumaric acid
SA: syringic acid
