# Supplementary material for: Internalization and accumulation of model lignin breakdown products in bacteria and fungi
Source: Biotechnol Biofuels. 2019 Jul 3;12:175. doi: 10.1186/s13068-019-1494-8 (PMC6607601; doi:10.1186/s13068-019-1494-8)
Supplement: Supplementary file 1 — Additional file 1: Figure S1. A. Representative mass spectra (QToF-MS) for each uptake compound (4-HBA, VA, SA, CA, and GGE) in P. chrysosporium lysates. DMSO vehicle control mass spectra are shown at the retention time for each compound of interest for peak comparison. Red arrows mark the integrated peak for results in B (4 h). B. Ion counts for each compounds in P. chrysosporium cell lysates from 3 biological replicates. Error bars are standard deviation. C. Ion counts for spiked P. chrysosporium lysate with indicated compounds at a concentration of 0.005 or 0.0025 mM. Spikes were used to calculate the relative lysate concentrations in Fig. 1 from ion counts in Fig. S1B. D. Representative mass spectra for protocatechuate (GGE metabolite) in P. chrysosporium lysates at 4 h. DMSO vehicle control mass spectra shown at the same retention time for peak comparison. Black arrows mark the integrated metabolite peak for Fig. 1c. [file 13068_2019_1494_MOESM1_ESM.pdf]

A

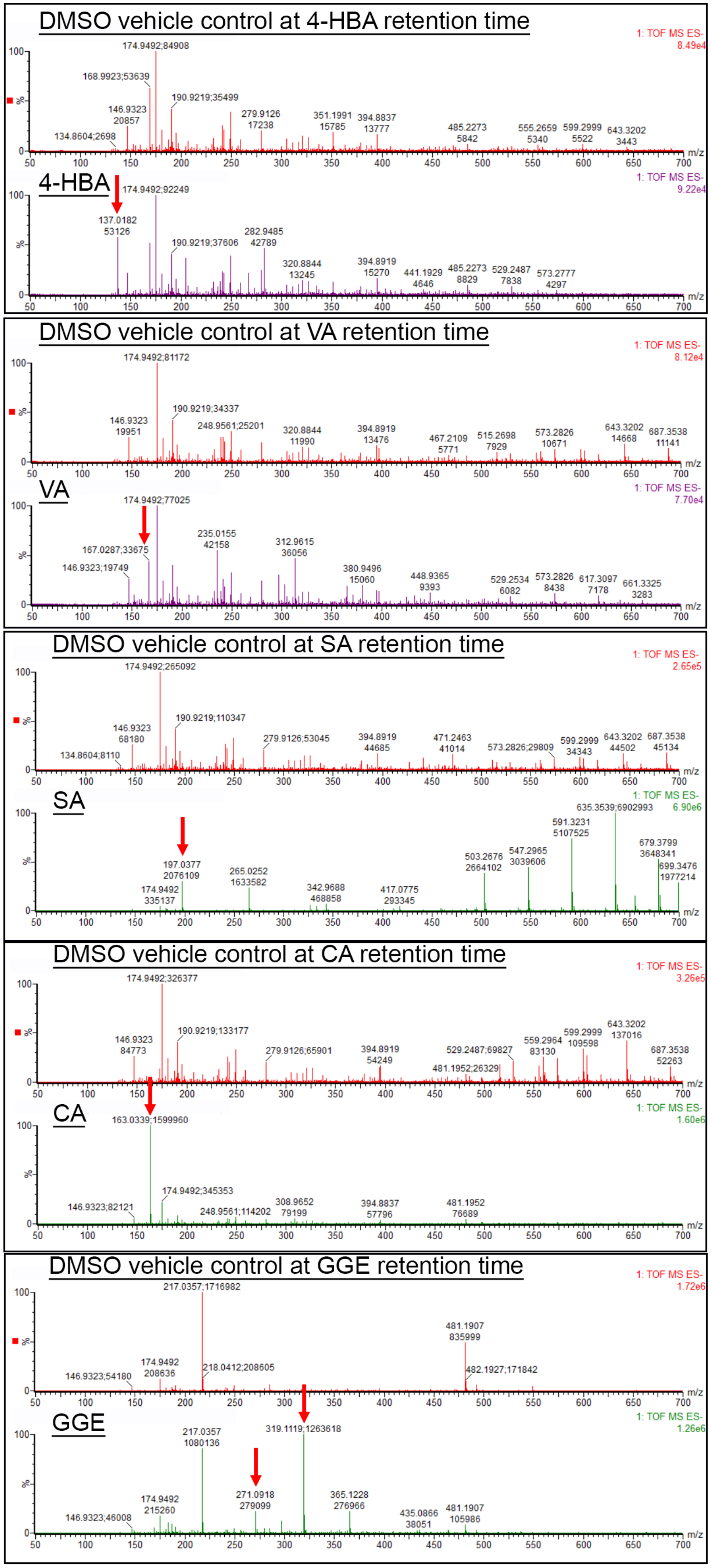

B

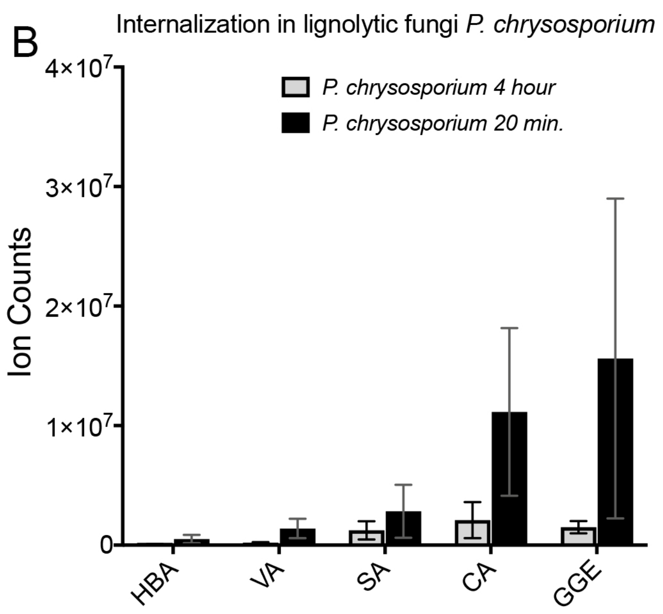

C

	P. chrysosporium 0.005mM Spike	*P. chrysosporium* 0.0025mM Spike
4-HBA	--	20540
VA	--	49045
SA	270974	--
CA	2660226	--
GGE	707870	--

D

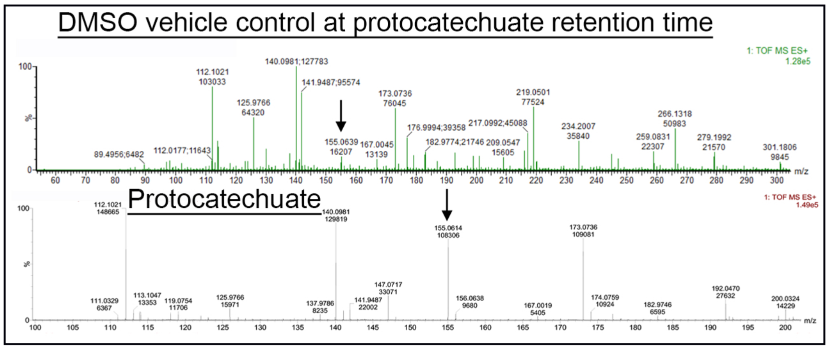

Fig. S1: A. Representative mass spectra (QToF-MS) for each uptake compound (4-HBA, VA, SA, CA, and GGE) in *P. chrysosporium* lysates. DMSO vehicle control mass spectra are shown at the retention time for each compound of interest for peak comparison. Red arrows mark the integrated peak for results in B (4 hours). B. Ion counts for each compound in *P. chrysosporium* cell lysates from 3 biological replicates. Error bars are standard deviation. C. Ion counts for spiked *P. chrysosporium* lysate with indicated compounds at a concentration of 0.005 or 0.0025mM. Spikes were used to calculate the relative lysate concentrations in Fig. 1 from ion counts in Fig. S1B. D. Representative mass spectra for protocatechuate (GGE metabolite) in *P. chrysosporium* lysates at 4 hours. DMSO vehicle control mass spectra shown at the same retention time for peak comparison. Black arrows mark the integrated metabolite peak for Fig. 1C.
