# Supplementary figures and images for: Internalization and accumulation of model lignin breakdown products in bacteria and fungi
Source: Biotechnol Biofuels. 2019 Jul 3;12:175. doi: 10.1186/s13068-019-1494-8 (PMC6607601; doi:10.1186/s13068-019-1494-8)

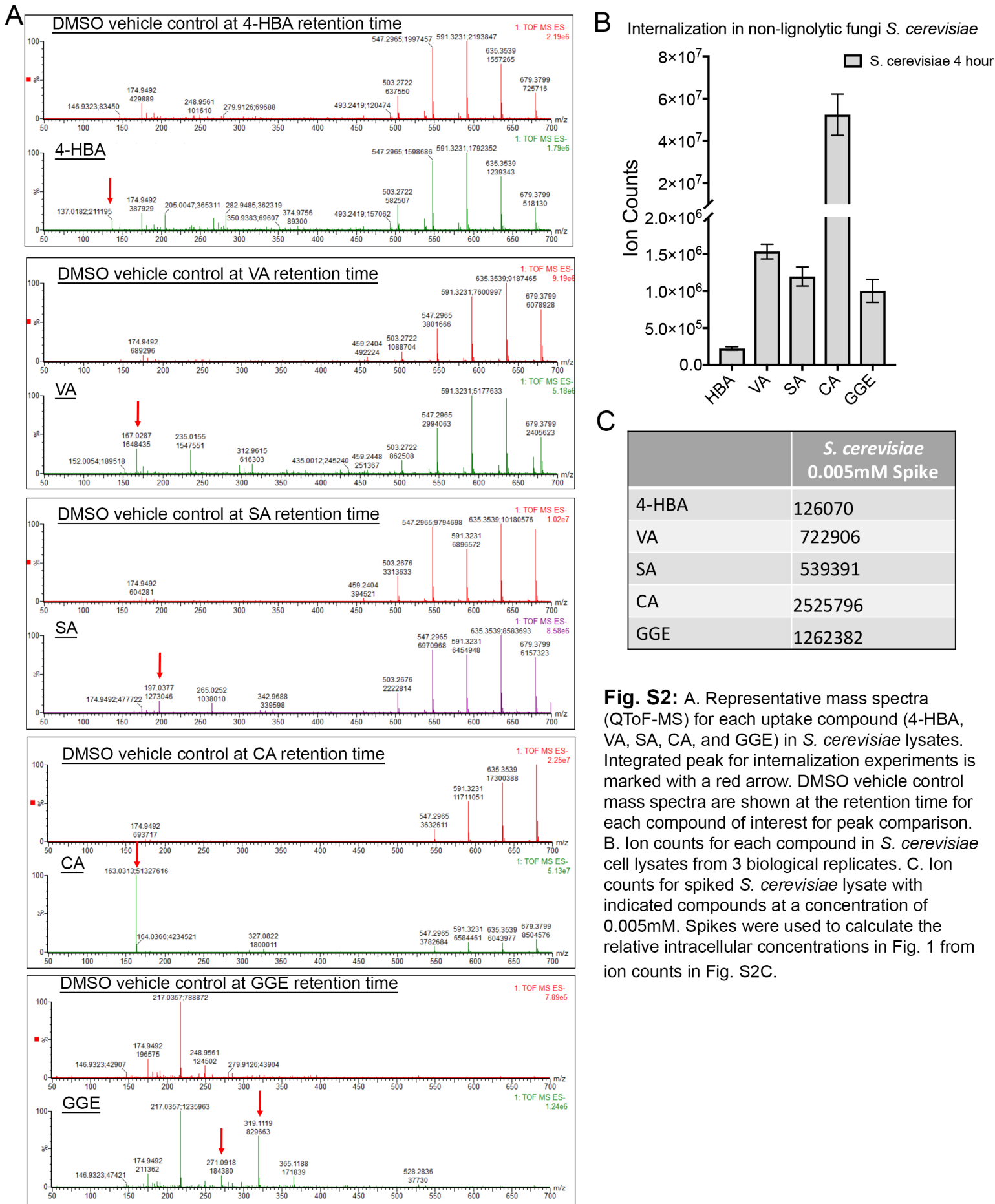

Supplement: Supplementary file 2 — Additional file 2: Figure S2. A. Representative mass spectra (QToF-MS) for each uptake compound (4-HBA, VA, SA, CA, and GGE) in S. cerevisiae lysates. Integrated peak for internalization experiments is marked with a red arrow. DMSO vehicle control mass spectra are shown at the retention time for each compound of interest for peak comparison. B. Ion counts for each compounds in S. cerevisiae cell lysates from 3 biological replicates. C. on counts for spiked S. cerevisiae lysate with indicated compounds at a concentration of 0.005 mM. Spikes were used to calculate the relative intracellular concentration in Fig. 1 from ion counts in Fig. S2C. [file 13068_2019_1494_MOESM2_ESM.pdf]

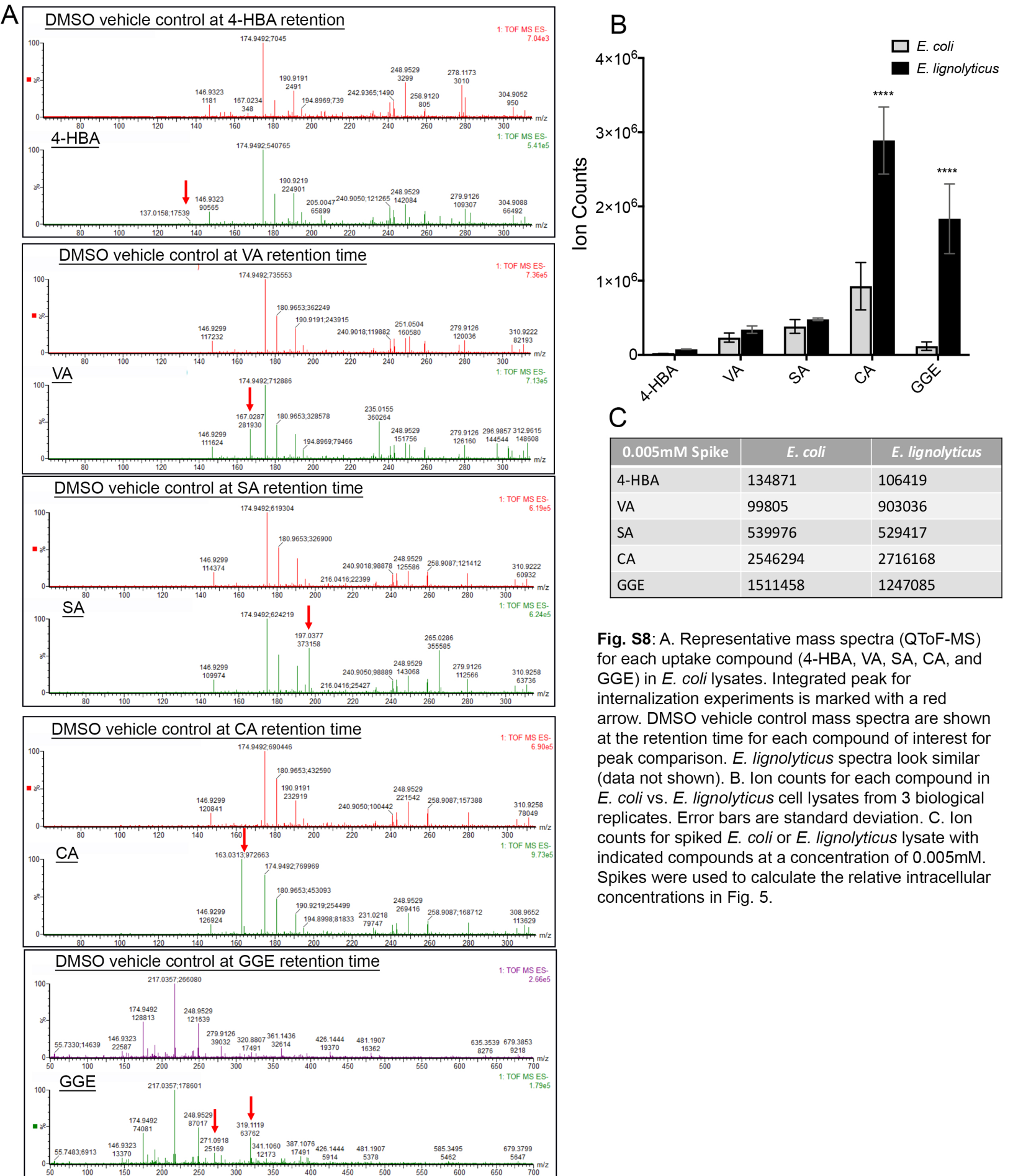

Supplement: Supplementary file 8 — Additional file 8: Figure S8. A. Representative mass spectra (QToF-MS) for each uptake compound (4-HBA, VA, SA, CA, and GGE) in E. coli lysates. Integrated peak for internalization experiments is marked with a red arrow. DMSO vehicle control mass spectra are shown at the retention time for each compound of interest for peak comparison. E. lignolyticus spectra look similar (data not shown). B. ion counts for each compound in E. coli vs. E. lignolyticus cell lysates from 3 biological replicates. Error bars are standard deviation. C. Ion counts for spiked E. coli or E. lignolyticus lysate with indicated compounds at a concentration of 0.005 mM. Spikes were used to calculate the relative intracellular concentrations in Fig. 5. [file 13068_2019_1494_MOESM8_ESM.pdf]
