# Supplementary material for: Internalization and accumulation of model lignin breakdown products in bacteria and fungi
Source: Biotechnol Biofuels. 2019 Jul 3;12:175. doi: 10.1186/s13068-019-1494-8 (PMC6607601; doi:10.1186/s13068-019-1494-8)
Supplement: Supplementary file 3 — Additional file 3: Figure S3. A monofunctionalized vanillic acid analog, where the methoxy group in vanillic acid was replaced by the propynyloxy group and the p-hydroxy remained unfunctionalized, (Fig. S3, box) would perhaps be a better vanillic acid mimic by preserving the hydrogen bonding of the p-hydroxy. [file 13068_2019_1494_MOESM3_ESM.pdf]

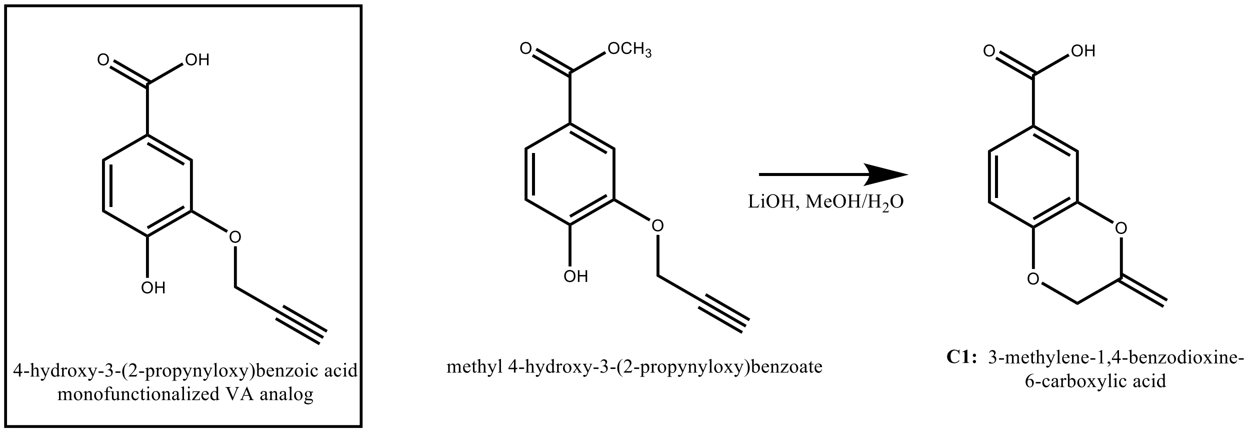

Fig. S3: A monofunctionalized vanillic acid analog, where the methoxy group in vanillic acid was replaced by the propynyloxy group and the p-hydroxy remained unfunctionalized, (Fig. S3, box) would perhaps be a better vanillic acid mimic by preserving the hydrogen bonding of the p-hydroxy. However, although the monofunctionalized methyl benzoate could be synthesized and purified, deprotection of the methyl 4-hydroxy-3-(2-propynyloxy)benzoate did not yield the targeted monofunctionalized vanillic acid analog but instead resulted in cyclization yielding the cyclic compound **C1,** 2-methylene-1,4-benzodioxine-6-carboxylic acid (Fig. S3, right side). A variety of reaction and work-up conditions were explored, but cyclization occurred invariably, and NMR analysis showed that the cyclization was occurring prior to work-up. Similar cyclizations of propynylated catechol-derivatives have been previously reported in the literature (1, 2), although promoted by metal catalysts. In this case, no catalyst was added, and it was observed that prior to deprotection of the carboxylic acid the compound was stable. Although the synthesis was performed with standard purity reagents and glassware was cleaned by typical methods, it is possible that adventitious trace metals catalyzed the cyclization. Previous reports have not included cyclizations yielding 6-carboxylic acid functionalized benzodioxins, as reported here. This reaction could provide an entry into biologically or pharmaceutically interesting 6-functionalized 1,4-benzodioxins (3).

The monofunctionalized methyl 4-hydroxy-3-(2-propynyloxy)benzoate was a co-product in the synthesis of difunctionalized **fVA-CH_3** (see Methods section). Isolated yield of the monofunctionalized product was 11% (143.1mg, 0.69mmol).

C1 (2-methylene-1,4-benzodioxine-6-carboxylic acid): Methyl 4-hydroxy-3-(2-propynyloxy)benzoate (143.1mg, 0.69mmol) was placed in a 2-neck round-bottom flask fitted with a reflux condenser. The assembly was purge/vac'd x3 with argon. Meanwhile, 20mL of ethanol and 5mL of water were combined and degassed, and then 5mL of the mixture was added to the reaction flask via syringe. LiOH (185mg, 7.7mmol) was added against a positive flow of argon, and the reaction mixture was refluxed under argon for 14 hours. The reaction mixture was transferred to a 50mL roundbottom with 20mL of MeOH and rotovapped to yield a white slurry. 1M HCl was added until the solution cleared, and then copious amounts of white precipitate formed on further acidification (final pH<3). The white precipitate is filtered and washed thoroughly with water, then dried overnight. The product is recrystallized from hot ethanol (cool to RT then recrystallize at 4 ˚C) to yield the cyclic product **C1** in 77% yield (103mg, 0.53mmol). ^{1}H NMR (500MHz, d_6-DMSO): δ 12.85 (s, 1H, COOH), 7.51 (dd, J = 8.4Hz, 2.0Hz, 1H, CH_{Ar}), 7.49 (d, J = 2.0Hz, 1H, CH_{Ar}), 7.04 (d, J = 8.4Hz, 1H, CH_{Ar}), 4.80 (d, J = 1.8Hz, 1H, $C=CH_2$), 4.69 (s, 2H, $O-CH_2$), 4.61 (d, J = 1.8Hz, 1H, $C=CH_2$)

1. Chowdhury C, Kundu NG. 1996. A totally stereoselective synthesis of (Z)-2-arylidene-1,4-benzodioxanes using palladium-copper catalysis. Chemical Communications doi:DOI 10.1039/cc9960001067:1067-1068.
2. Gabriele B, Salerno G, Veltri L, Mancuso R, Li ZY, Crispini A, Bellusci A. 2006. A new synthesis of 2,3-dihydrobenzo[1,4]dioxine and 3,4-dihydro-2H-benzo[1,4]oxazine derivatives by tandem palladium-catalyzed oxidative aminocarbonylation - Cyclization of 2-prop-2-ynyloxyphenols and 2-prop-2-ynyloxyanilines. Journal of Organic Chemistry 71:7895-7898.
3. Achari B, Mandal SB, Dutta PK, Chowdhury C. 2004. Perspectives on 1,4-benzodioxins, 1,4-benzoxazines and their 2,3-dihydro derivatives. Synlett doi:10.1055/s-2004-834816:2449-2467.
