# Supplementary material for: Internalization and accumulation of model lignin breakdown products in bacteria and fungi
Source: Biotechnol Biofuels. 2019 Jul 3;12:175. doi: 10.1186/s13068-019-1494-8 (PMC6607601; doi:10.1186/s13068-019-1494-8)
Supplement: Supplementary file 4 — Additional file 4: Figure S4. Table summarizing single cell fluorescence microscopy results. [file 13068_2019_1494_MOESM4_ESM.pdf]

Figure S4: Table summarizing single cell fluorescence microscopy results.

		Biological Replicate	Total # Cells	# + Cells	% + Cells	Mean Intensity	Std Dev Mean Intensity
E. coli	*DMSO*	Rep 1	2599	6	0%	n/a	n/a
		Rep 2	4355	12	0%	n/a	n/a
		Rep3	1995	7	0%	n/a	n/a
	4-HBA analog	Rep 1	1212	43	4%	718.6	397.1
		Rep 2	2698	54	2%	760.5	355
		Rep3	1408	75	5%	779.3	483.1
	VA analog	Rep 1	745	241	32%	1853.8	1594.9
		Rep 2	2062	225	11%	1419.1	1367.4
		Rep3	1109	295	27%	1802.7	1262
E. lignolyticus	*DMSO*	Rep 1	577	6	1%	n/a	n/a
		Rep 2	485	8	2%	n/a	n/a
		Rep3	524	6	1%	n/a	n/a
	4-HBA analog	Rep 1	1184	257	22%	138.5	99
		Rep 2	854	517	61%	886.2	1821.7
		Rep3	661	316	48%	306.7	549.9
	VA analog	Rep 1	888	478	54%	1868	2141.7
		Rep 2	597	368	62%	959.2	1510.2
		Rep3	1305	802	61%	605.1	369.4
S. cerevisiae	*DMSO*	Rep 1	130	10	8%	n/a	n/a
		Rep 2	169	0	0%	n/a	n/a
		Rep3	252	3	1%	n/a	n/a
	4-HBA analog	Rep 1	118	23	19%	1187.8	592.7
		Rep 2	586	67	11%	703.5	307.6
		Rep3	215	53	25%	663.9	169.4
	VA analog	Rep 1	532	271	51%	2650	2208.5
		Rep 2	400	117	29%	1977.8	1546.6
		Rep3	191	124	65%	2934.2	1756.3
P. chysosporium	*DMSO*	Rep 1	89	7	8%	n/a	n/a
		Rep 2	36	0	0%	n/a	n/a
		Rep3	59	8	14%	n/a	n/a
	4-HBA analog	Rep 1	183	12	7%	716.9	117.1
		Rep 2	153	0	0%	0	0
		Rep3	244	94	39%	1126.9	454.1
	VA analog	Rep 1	124	18	15%	2931.3	1528.9
		Rep 2	55	22	40%	4622	2758.6
		Rep3	105	90	86%	3134.3	1281.5
