# Supplementary material for: Internalization and accumulation of model lignin breakdown products in bacteria and fungi
Source: Biotechnol Biofuels. 2019 Jul 3;12:175. doi: 10.1186/s13068-019-1494-8 (PMC6607601; doi:10.1186/s13068-019-1494-8)
Supplement: Supplementary file 5 — Additional file 5: Figure S5. Single cell AlexaFluor™ 647 intensities of A. S. cerevisiae B. P. chrysosporium V. E. coli, and D. E. lignolyticus. Thin red line is the mean and red whiskers mark the standard deviation. Intensities shown are from the cells deemed positive in three biological replicates based on stringent thresholds to minimize the moderate levels of autofluorescence (see “Materials and Methods” for detailed description). Mean intensity data is presented raw and does not compensate for the vanillic acid analog being difunctionalized. [file 13068_2019_1494_MOESM5_ESM.pdf]

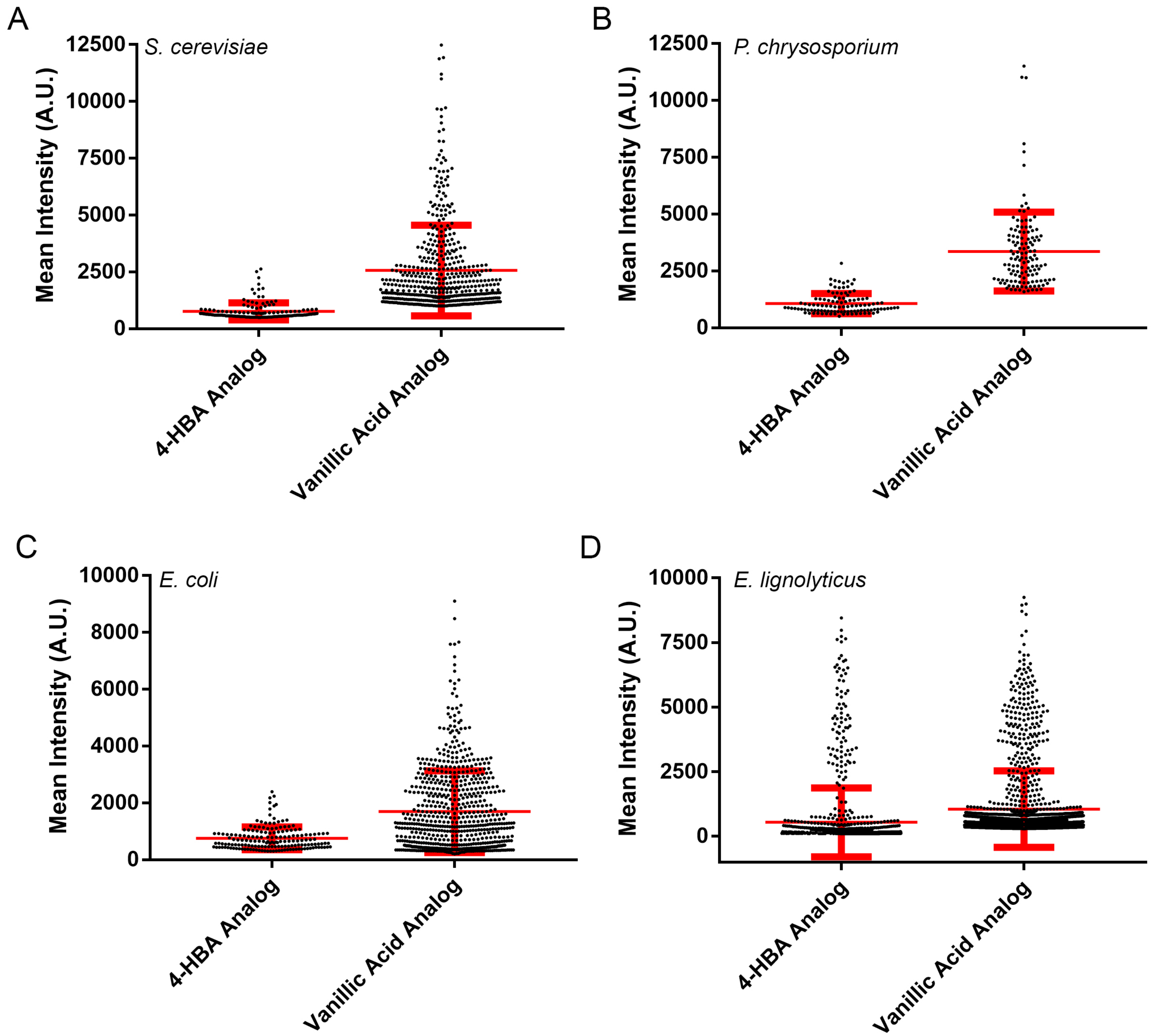

Fig. S5. Single cell Alexa Fluor™ 647 intensities of A. *S. cerevisiae* B. *P. chrysosporium*, C. *E. coli,* and D. *E. lignolyticus*. Thin red line is the mean and red whiskers mark the standard deviation. Intensities shown are from the cells deemed positive in three biological replicates based on stringent thresholds to minimize the moderate levels of autofluorescence (see Methods for detailed description). Mean intensity data is presented raw and does not compensate for the vanillic acid analog being difunctionalized.
