# Supplementary material for: Internalization and accumulation of model lignin breakdown products in bacteria and fungi
Source: Biotechnol Biofuels. 2019 Jul 3;12:175. doi: 10.1186/s13068-019-1494-8 (PMC6607601; doi:10.1186/s13068-019-1494-8)
Supplement: Supplementary file 6 — Additional file 6: Figure S7. Manganese peroxidase assay measuring absorbance at 270 nm of concentrated Kirk’s mineral media following 5 days of P. chrysosporium growth. Media contained either 2 mg/mL microcrystalline cellulose (blue line) or 10 mg/mL glucose (red line) during the 5 day growth period. N = 3 biological replicates (each point is average of 3 media measurements). [file 13068_2019_1494_MOESM6_ESM.pdf]

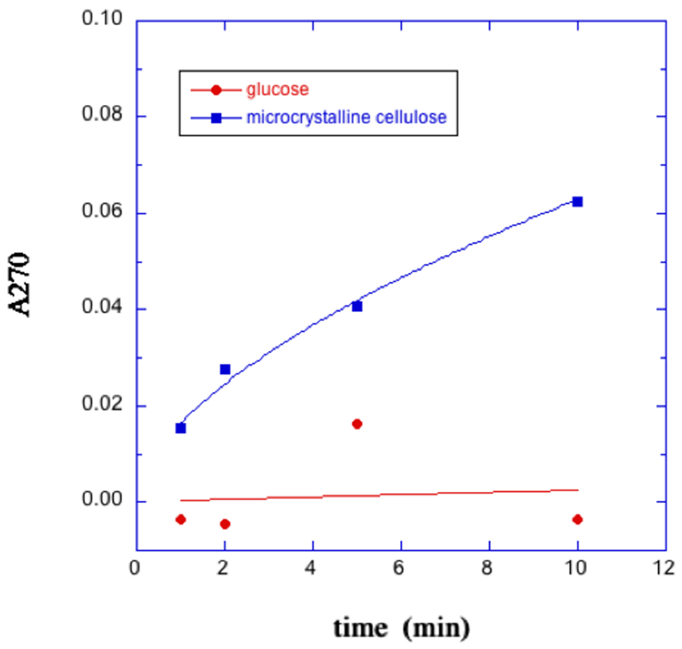

Figure S7. Manganese peroxidase assay measuring absorbance at 270nm of concentrated Kirk's mineral media following 5 days of *P. chrysosporium* growth. Media contained either 2mg/mL microcrystalline cellulose (blue line) or 10mg/mL glucose (red line) during the 5 day growth period. N=3 biological replicates (each point is average of 3 media measurements).
