# Supplementary material for: Internalization and accumulation of model lignin breakdown products in bacteria and fungi
Source: Biotechnol Biofuels. 2019 Jul 3;12:175. doi: 10.1186/s13068-019-1494-8 (PMC6607601; doi:10.1186/s13068-019-1494-8)
Supplement: Supplementary file 7 — Additional file 7: Figure S6. A. ASAP-MS representative spectra of S. cerevisiae lysates incubated with vanillic acid for 1, 2, 3, or 17 h. Blue arrow indicates integrated peak of interest for results displayed in Fig. 4c. B. ASAP-MS representative chromatogram of P. chrysosporium lysates incubated with vanillic acid for 1, 2, 4, or 24 h. Orange arrow indicates integrated peak of interest for results displayed in Fig. 4b. C. ASAP-MS representative chromatogram of P. chrysosporium lysates incubated with 13C labeled vanillic acid for 1, 2, 4, or 24 h. Red arrow indicates integrated peak of interest for results displayed in Fig. 4b. D. ASAP-MS representative chromatogram of P. chrysosporium lysates incubated with vanillic acid for 5, 15, and 60 min. Orange arrow indicates integrated peak of interest for results displayed in Fig. 4b. [file 13068_2019_1494_MOESM7_ESM.pdf]

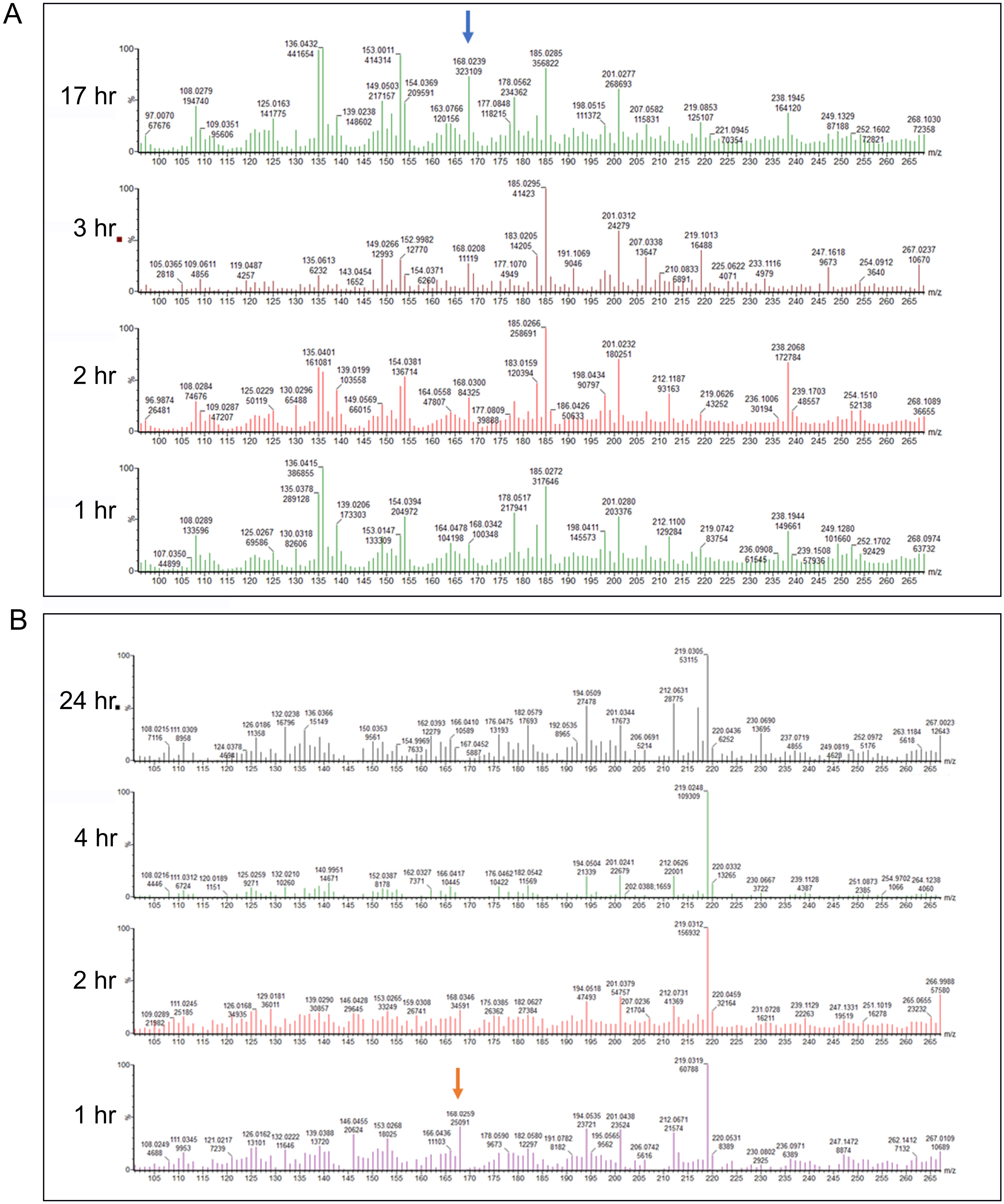

A
17 hr
136.0432 441654
153.0011 414314
168.0239 323109
185.0285 356822
201.0277 268693
108.0279 194740
149.0503 217157
154.0369 209591
178.0562 234362
97.0070 67676
109.0351 95606
125.0163 141775
139.0238 148602
163.0766 120156
177.0848 118215
198.0515 111372
207.0582 115831
219.0853 125107
221.0945 70354
238.1945 164120
249.1329 87188
252.1602 72821
268.1030 72358
3 hr
185.0295 41423
201.0312 24279
183.0205 14205
219.1013 16488
149.0266 12993
152.9982 12770
105.0365 2818
109.0611 4856
119.0487 4257
135.0613 6232
143.0454 1652
154.0371 6260
168.0208 11119
177.1070 4949
191.1069 9046
207.0338 13647
210.0833 6891
225.0622 4071
233.1116 4979
247.1618 9673
254.0912 3640
267.0237 10670
2 hr
185.0266 258691
201.0232 180251
238.2068 172784
135.0401 161081
139.0199 103558
154.0381 136714
183.0159 120394
96.9874 26481
108.0284 74676
109.0287 47207
125.0229 50119
130.0296 65488
149.0569 66015
164.0558 47807
168.0300 84325
177.0809 39888
186.0426 50633
198.0434 90797
212.1187 93163
219.0626 43252
236.1006 30194
239.1703 48557
254.1510 52138
268.1089 36655
1 hr
136.0415 386855
135.0378 289128
185.0272 317646
178.0517 217941
201.0280 203376
139.0206 173303
154.0394 204972
108.0289 133596
107.0350 44899
125.0267 69586
130.0318 82606
153.0147 133309
164.0478 104198
168.0342 100348
198.0411 145573
212.1100 129284
219.0742 83754
236.0908 61545
238.1944 149661
239.1508 57936
249.1280 101660
252.1702 92429
268.0974 63732
m/z
B
24 hr
219.0305 53115
212.0631 28775
194.0509 27478
201.0344 17673
182.0579 17693
108.0215 7116
111.0309 8958
124.0378 4694
126.0186 11358
132.0238 16796
136.0366 15149
150.0353 9561
154.9969 7633
162.0393 12279
166.0410 10589
167.0452 5887
176.0475 13193
192.0535 8965
206.0691 5214
220.0436 6252
230.0690 13695
237.0719 4855
249.0819 4628
252.0972 5176
263.1184 5618
267.0023 12643
4 hr
219.0248 109309
108.0216 4446
111.0312 6724
120.0189 1151
125.0259 9271
132.0210 10260
140.9951 14671
152.0387 8178
162.0327 7371
166.0417 10445
176.0462 10422
182.0542 11569
194.0504 21339
201.0241 22679
202.0388 1659
212.0626 22001
220.0332 13265
230.0667 3722
239.1128 4387
251.0873 2385
254.9702 1066
264.1238 4060
2 hr
219.0312 156932
109.0289 21982
111.0245 25185
126.0168 34935
129.0181 36011
139.0290 30857
146.0428 29645
153.0265 33249
159.0308 26741
168.0346 34591
175.0385 26362
182.0627 27384
194.0518 47493
201.0379 54757
207.0236 21704
212.0731 41369
220.0459 32164
231.0728 16211
239.1129 22263
247.1331 19519
251.1019 16278
265.0655 23232
266.9988 57580
1 hr
219.0319 60788
168.0259 25091
108.0249 4688
111.0345 9953
121.0217 7239
126.0162 13101
132.0222 11646
139.0388 13720
146.0455 20624
153.0268 18025
166.0436 11103
178.0590 9673
182.0580 12297
191.0782 8182
194.0535 23721
195.0565 9562
201.0438 23524
206.0742 5616
212.0671 21574
220.0531 8389
230.0802 2925
236.0971 6389
247.1472 8874
262.1412 7132
267.0109 10689
m/z

C

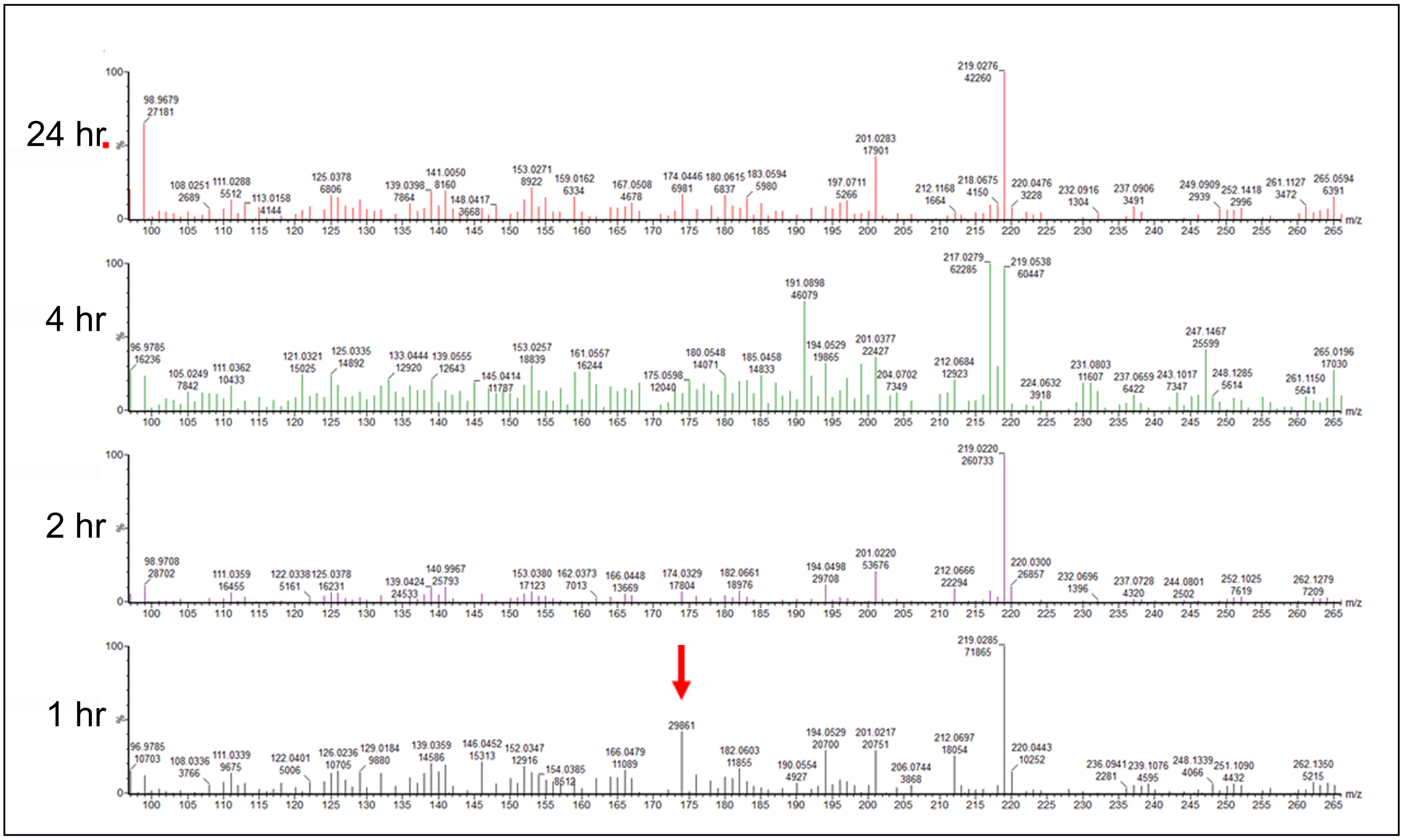

D

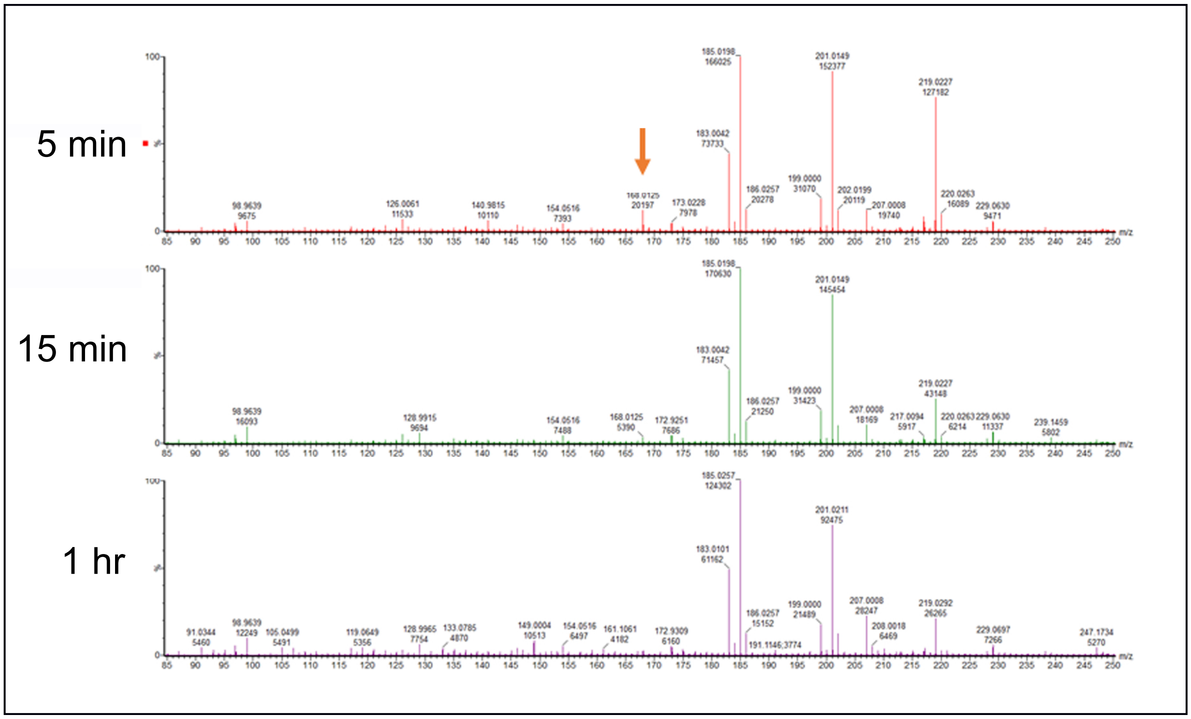

Fig. S6: A. ASAP-MS representative spectra of *S. cerevisiae* lysates incubated with vanillic acid for 1, 2, 3, or 17 hours. Blue arrow indicates integrated peak of interest for results displayed in Fig. 4C. B. ASAP-MS representative chromatogram of *P. chrysosporium* lysates incubated with vanillic acid for 1, 2, 4, or 24 hours. Orange arrow indicates integrated peak of interest for results displayed in Fig. 4B. C. ASAP-MS representative chromatogram of *P. chrysosporium* lysates incubated with 13C labeled vanillic acid for 1, 2, 4, or 24 hours. Red arrow indicates integrated peak of interest for results displayed in Fig. 4B. D. ASAP-MS representative chromatogram of *P. chrysosporium* lysates incubated with vanillic acid for 5, 15, and 60 min. Orange arrow indicates integrated peak of interest for results displayed in Fig. 4B.
