# Supplementary material for: Internalization and accumulation of model lignin breakdown products in bacteria and fungi
Source: Biotechnol Biofuels. 2019 Jul 3;12:175. doi: 10.1186/s13068-019-1494-8 (PMC6607601; doi:10.1186/s13068-019-1494-8)
Supplement: Supplementary file 9 — Additional file 9: Figure S9. A. Representative mass spectra for GGE metabolites (guiacol, protocatechuate, and 2-(2-methoxyphenoxy)-1,3-propanediol) from E. lignolyticus lysates grown in minimal media and incubated with DMSO or GGE for 4 h. Integrated peak for each (from left to right in order listed above) is marked with a red arrow. B. Ion counts for integrated peaks indicated with arrows in A. Gray bars are ion counts of each compound for DMSO lysate, and black bars are ion counts of each compound for GGE lysates. 1 biological replicates is shown. C. Representative mass spectra for GGE metabolics (guiacol, protocatechuate, and 2-(2-methoxyphenoxy)-1,3-propanediol) from E. lignolyticus lysates grown in nutrient broth and incubated with DMSO or GGE for 4 h. Integrated peak for protocatechuate is marked with a red arrows. D. Average ion counts for integrated protocatechuate peak indicated with arrows in C. 3 biological replicates are shown, error bars are standard deviation. Gray bars are ions counts for DMSO lysate and black bars are ion counts for the GGE lysates. [file 13068_2019_1494_MOESM9_ESM.pdf]

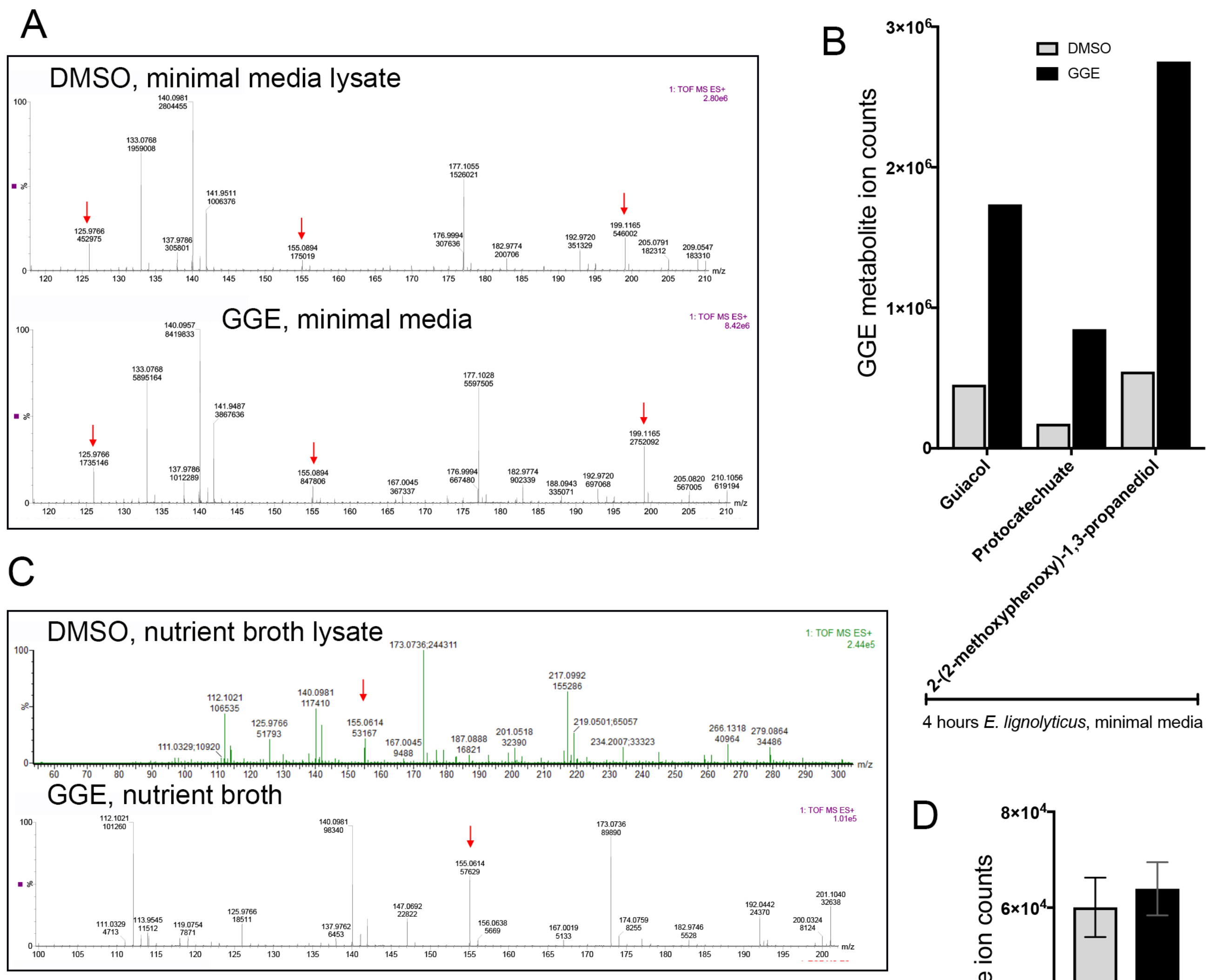

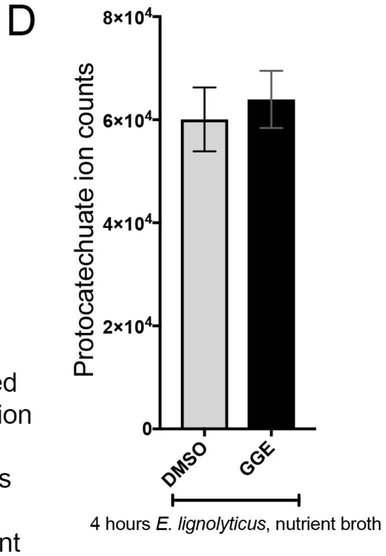

Fig. S9: A. Representative mass spectra for GGE metabolites (guiacol, protocatechuate, and 2-(2-methoxyphenoxy)-1,3-propanediol) from *E. lignolyticus* lysates grown in minimal media and incubated with DMSO or GGE for 4 hours. Integrated peak for each (from left to right in order listed above) is marked with a red arrow. B. Ion counts for integrated peaks indicated with arrows in A. Gray bars are ion counts of each compound for DMSO lysate, and black bars are ion counts of each compound for GGE lysates. 1 biological replicate is shown. C. Representative mass spectra for GGE metabolites (guiacol, protocatechuate, and 2-(2-methoxyphenoxy)-1,3-propanediol) from *E. lignolyticus* lysates grown in nutrient broth and incubated with DMSO or GGE for 4 hours. Integrated peak for protocatechuate is marked with a red arrow. D. Average ion counts for integrated protocatechuate peak indicated with arrows in C. 3 biological replicates are shown, error bars are standard deviation. Gray bars are ion counts for DMSO lysate, and
