# Supplementary material for: Internalization and accumulation of model lignin breakdown products in bacteria and fungi
Source: Biotechnol Biofuels. 2019 Jul 3;12:175. doi: 10.1186/s13068-019-1494-8 (PMC6607601; doi:10.1186/s13068-019-1494-8)
Supplement: Supplementary file 10 — Additional file 10: Figure S10. Area (pX2) of E. lignolyticus cells following internalization of 4-HBA or VA for 4 h. Cells areas were measures using DAPI signal. ****p < 0.0001 by Kruskal–Wallis test. Red bars mark the average and whiskers are the standard deviation. N = 3 biological replicates. B. Aspect ratio measurements of E. lignolyticus cells following internalization of 4-HBA or VA for 4 h. Cells aspect ratios were measured using the longest and shortest axis using DAPI. signal. ****p < 0.0001 by Kruskal-Wallis test. Red bars mark the average and whiskers are the standard deviation. N = 3 biological replicates. [file 13068_2019_1494_MOESM10_ESM.pdf]

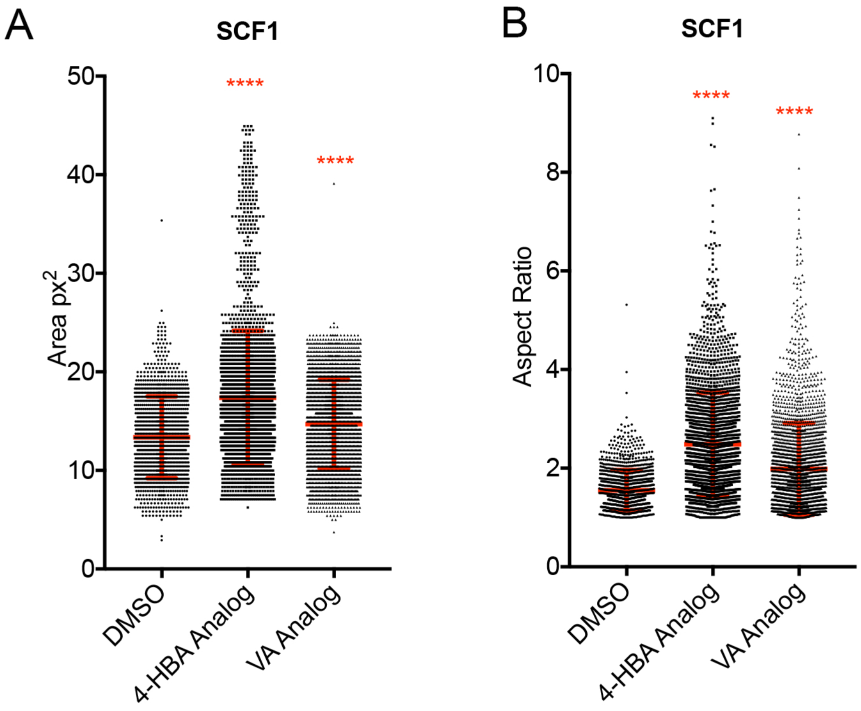

Fig. S10. A. Area (px$^{2)}$ of *E. lignolyticus* cells following internalization of 4-HBA or VA for 4 hours. Cells areas were measured using DAPI signal. ****p<0.0001 by Kruskal-Wallis test. Red bars mark the average and whiskers are the standard deviation. N=3 biological replicates. B. Aspect ratio measurements of *E. lignolyticus* cells following internalization of 4-HBA or VA for 4 hours. Cells aspect ratios were measured using the longest and shortest axis using DAPI signal. ****p<0.0001 by Kruskal-Wallis test. Red bars mark the average and whiskers are the standard deviation. N=3 biological replicates.
