# Supplementary material for: Internalization and accumulation of model lignin breakdown products in bacteria and fungi
Source: Biotechnol Biofuels. 2019 Jul 3;12:175. doi: 10.1186/s13068-019-1494-8 (PMC6607601; doi:10.1186/s13068-019-1494-8)
Supplement: Supplementary file 11 — Additional file 11: Figure S11. A. LC/MS standard curve of 4-HBA prepared in methanol at 0.001, 0.005, 0.01, 0.05, 0.1 mM. b. LC/MS standard curve of VA prepared in methanol at 0.001, 0.005, 0.01, 0.05, and 0.1 mM. C. LC/MS standard curve of CA prepared in methanol at 0.001, 0.005, 0.01, 0.05, 0.1, 0.25, 0.5, 0.8, and 1 mM. D. LC/MS standard curve of GGE prepared in methanol at 0.005, 0.01, 0.05, and 0.1 mM. E. LC/MS standard curve of SA prepared in 10% methanol, 90% PBS at 0.01, 0.02, and 0.05 mM. [file 13068_2019_1494_MOESM11_ESM.pdf]

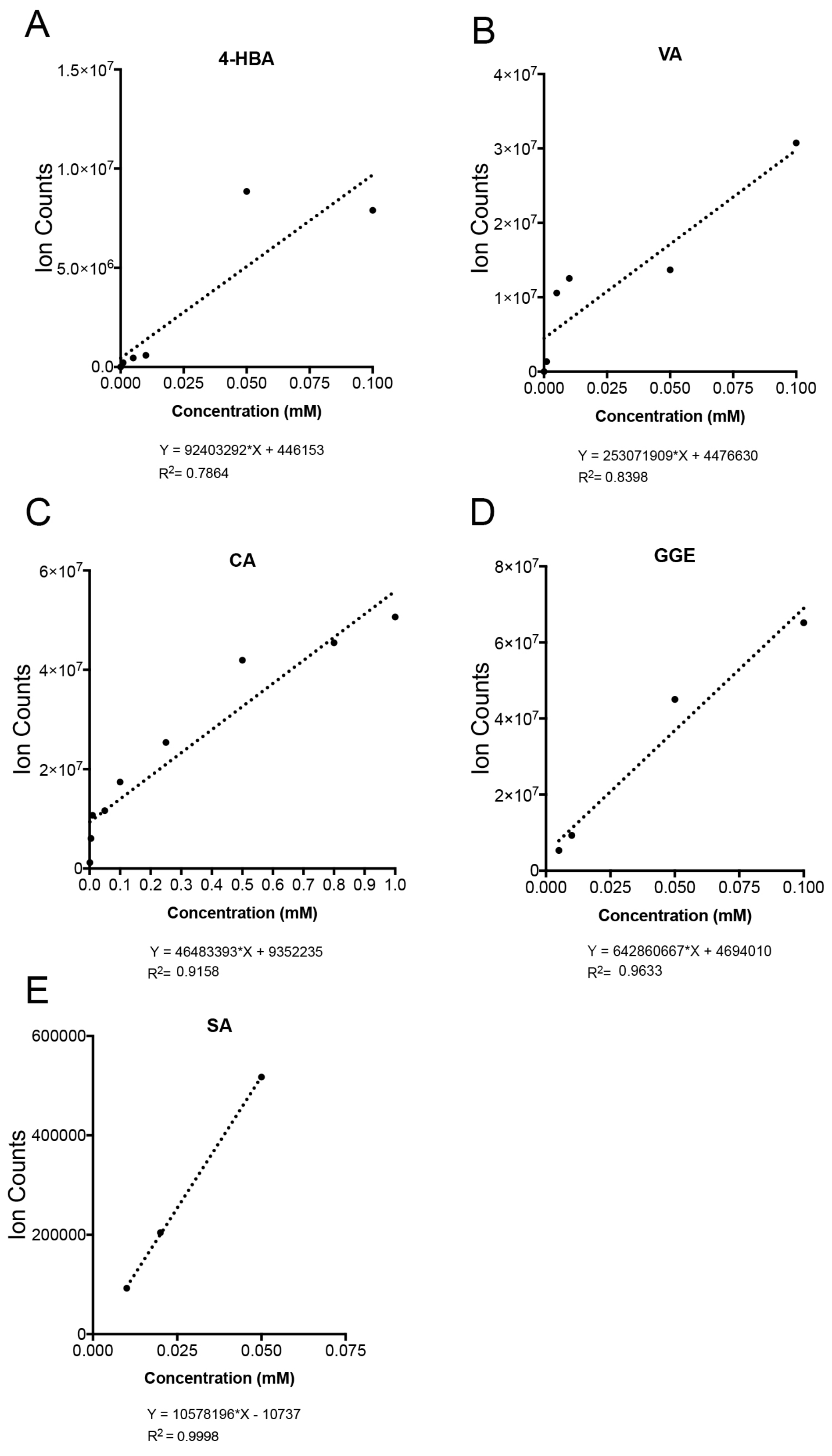

Fig. S11: A. LC/MS standard curve of 4-HBA prepared in methanol at 0.001, 0.005, 0.01, 0.05, and 0.1 mM. B. LC/MS standard curve of VA prepared in methanol at 0.001, 0.005, 0.01, 0.05, and 0.1 mM. C. LC/MS standard curve of CA prepared in methanol at 0.001, 0.005, 0.01, 0.05, 0.1, 0.25, 0.5, 0.8, and 1mM. D. LC/MS standard curve of GGE prepared in methanol at 0.005, 0.01, 0.05, and 0.1 mM. E. LC/MS standard curve of SA prepared in 10% methanol, 90% PBS at 0.01, 0.02, and 0.05mM.
